# Tumor suppressor activity of miR-451: Identification of CARF as a new target

**DOI:** 10.1038/s41598-017-18559-5

**Published:** 2018-01-10

**Authors:** Ling Li, Ran Gao, Yue Yu, Zeenia Kaul, Jia Wang, Rajkumar S. Kalra, Zhenya Zhang, Sunil C. Kaul, Renu Wadhwa

**Affiliations:** 1 Drug Discovery and Assets Innovation Lab, DBT-AIST International Laboratory for Advanced Biomedicine (DAILAB), DAICENTER, Biomedical Research Institute, National Institute of Advanced Industrial Science & Technology (AIST), Tsukuba, 305 8565 Japan; 2 Graduate School of Life and Environmental Sciences, University of Tsukuba, Tsukuba, Japan; 3 Institute of Laboratory Animal Science, Chinese Academy of Medical Science (CAMS) & Comparative Medicine Center, Peking Union Medical College (PUMC), Peking, China; 4 Department of Molecular Virology, Immunology and Medical Genetics, The Ohio State University, Columbus, Ohio, 43210 USA

## Abstract

microRNAs (miRs) have recently emerged as small non-coding regulators of gene expression. We performed a loss-of-function screening by recruiting retrovirus mediated arbitrary manipulation of genome coupled with escape of cells from 5-Aza-2′-deoxycytidine (5-Aza-dC)-induced senescence. miRNA pool from cells that emerged from 5-Aza-dC-induced senescence was subjected to miR-microarray analysis with respect to the untreated control. We identified miR-451 as one of the upregulated miRs and characterized its functional relevance to drug resistance, cell growth, tumor suppressor proteins p53 and pRb, and stress response. We report that miR-451 caused growth arrest in cells leading to their resistance to 5-Aza-dC-induced senescence. Decrease in cyclin D1, CDK4 and phosphorylated pRB supported the growth arrest in miR-451 transfected cells. We demonstrate that Collaborator of ARF (CARF) protein is a new target of miR-451 that intermediates its function in tumor suppressor and stress signaling.

## Introduction

Cancer also known as malignant neoplasm is a group of diseases involving abnormal cell growth and is extremely complex to understand and treat. According to World Health Organization (WHO), cancer caused 8.8 million deaths in 2015 and 30–50% of those could be prevented either by early diagnosis or improvement in therapeutic strategies. Current therapeutic solutions comprise surgical removal of detectable tumors and chemotherapy that is complicated by high toxicity, undesired secondary effects on normal body functions and drug resistance. Novel diagnostic markers and therapeutic drugs to improve the treatment are hence required.

MicroRNAs (miRNAs), single-stranded RNA of 18–24 nucleotides, first discovered in the early 1990s, have been recognized as noncoding regulators of gene functions including control of cell proliferation, differentiation, inflammation, stress response, apoptosis, carcinogenesis and metastasis^1–3^. Several miRNAs have been shown not only to act as tumor suppressors or oncogenes, but also serve as diagnostic markers important for the pathobiology of tumors^2,4^. Many miRNAs get downregulated in tumors and hence have been called tumor suppressors^2,4,5^. On the other hand, several miRNAs have been found to be upregulated in tumors and ascribed to upregulate oncogenic functions^2,5–7^.

Downregulation of miR-451 has been reported in a variety of tumors including glioma^8–11^, breast carcinoma^12^, gastrointestinal carcinoma^13^, non-small cell lung carcinoma (NSCLC)^14–17^, hepatoma^18–21^, nasopharyngeal^22^, esophageal^23,24^, bladder^25^, osteosarcoma^26,27^, epithelial ovarian^28^, renal^29^ and thyroid^30^ carcinomas. In several studies, it was shown to correlate with clinical stages of tumor including metastasis and poor response to neoadjuvant chemotherapy and recurrence^26,27^. Ling *et al*.^28^ examined the expression of miR-451 in 115 cancer and 34 normal ovarian tissues, and found correlation with clinicopathological factors and prognosis. Low level of miR-451 in ovarian cancer as compared with normal tissue was associated with advanced FIGO stage, higher serum CA125 expression level and lymph node metastasis. Furthermore, transfection of miR-451 mimics in ovarian cancer cells reduced their cell proliferation, promoted cell apoptosis, and inhibited cell invasion suggesting that miR-451 is a potential candidate for therapy^28^. Study showed that reduced miR-451 was significantly correlated with advanced clinical stage, metastasis and worse disease-free or overall survival in HCC tissues. Reconstitution of miR-451 caused growth arrest of HCC, increased their chemo- or radio-sensitivity and reversed epithelial to mesenchymal transition (EMT)^20,25^. Furthermore, decrease in Bcl-2, AKT and p-AKT expression resulted in increased apoptosis in miR-451 overexpressing esophageal carcinoma^23^.

miR-451 has been shown to target PSMB8 in renal cell carcinoma and lung cancer^16,29^; MIF, LKB1/AMPK, AMPK/mTOR and Fascin1 in thyroid, glioma, nasopharyngeal and gastric carcinomas^9,10,13,22,30^; IKK-β in HCC^19^; CXCL16 in osteosarcoma^26^; CDKN2D and MAP3K1 in esophageal carcinoma^24^; liver receptor homolog-1 (LRH-1) that plays crucial role in the onset and progression of many cancer types^31^; c-Myc/Erk1–2 and ATF2 in hepatocarcinoma^20,21^ and PI3K/Akt/mTOR in multiple myeloma^32^. Reconstitution of miRNA-451 inhibited cell cycle progression, cellular migration and the invasive ability of NSCLC cells. It was shown to target Ras-related protein 14 (RAB14)^15^ and serve as a novel therapeutic drug to treat NSCLC patients. Ectopic overexpression of miR-451 was shown to inhibit growth and induced apoptosis in A549 cells. It also sensitized them to cisplatin by inactivation of Akt signaling pathway^17^. miR-451 was shown to regulate the expression of multidrug resistance-1 gene. Transfection of the MCF-7/DOX-resistant cells with miR-451 sensitized them to DOX suggesting its implication for treatment of drug resistant cancer cells^12^.

In the present study, we used a bicistronic vector containing GFP reporter to arbitrarily induce miRs in human osteosarcoma^33^. Cells expressing such random library of miRs were subjected to 5-Aza-dC-induced senescence for 3–5 days. miR pool of cells that escaped senescence was subjected to miR-array analysis with respect to the control (untransfected) cells. Out of several upregulated miRs, we characterized the function of miR-451 in the present study. We report miR-451 caused growth arrest of cells that showed increase in p21^WAF1^, and decrease in CyclinD1, CDK4, phospho-pRB and E2F5 proteins. Expression and reporter assays demonstrated that these effects are mediated by targeting CARF.

## Results

### miR-451 possesses a tumor suppressor activity

**Figure 1:**
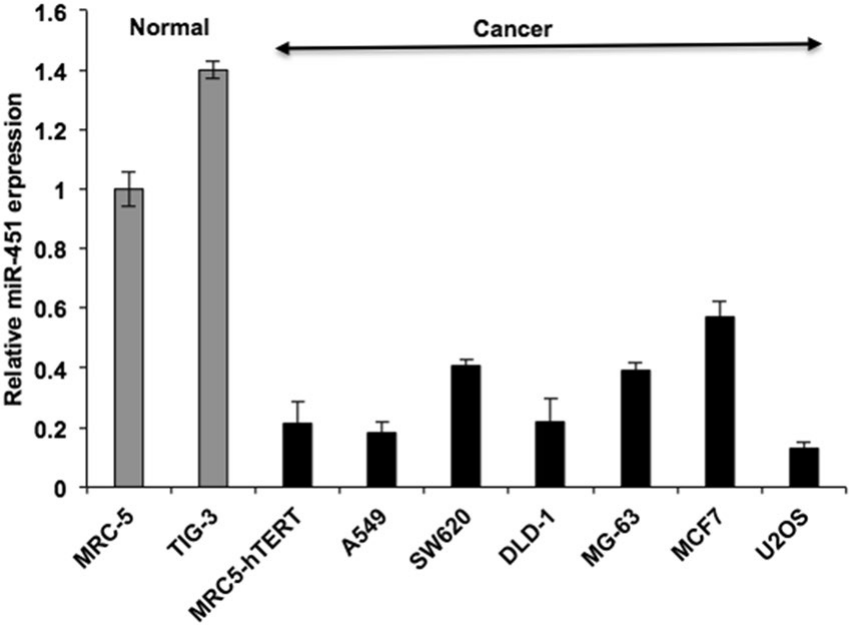
miR-451 is downregulated in cancer cells. Real-time PCR analysis of miR-451 in human normal and cancer cells showed its higher level of expression in normal cells. Cancer cells showed 2 to 10 fold less expression.

**Figure 2:**
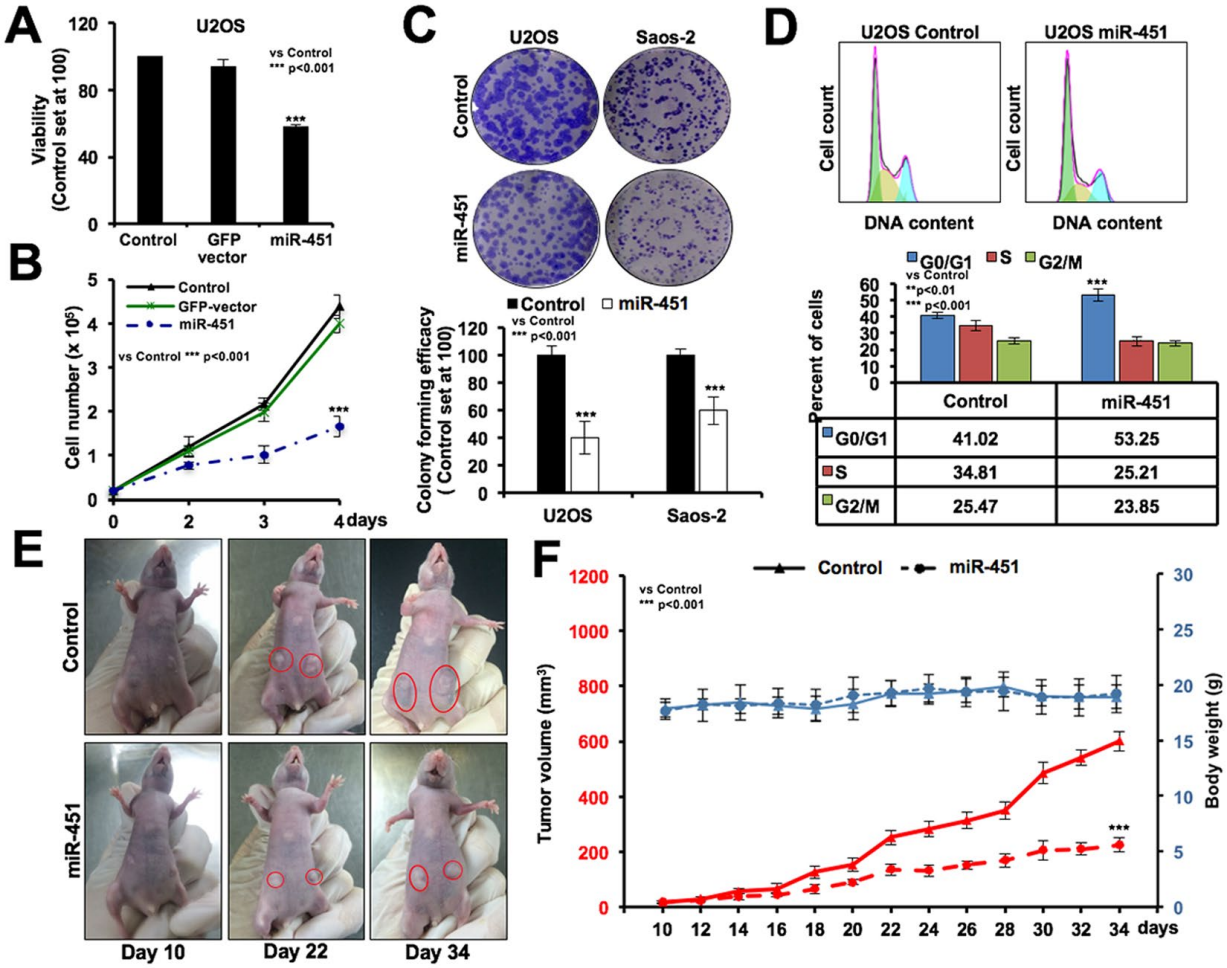
miR-451 overexpression inhibited cancer cell proliferation *in vitro* and *in vivo*. Viability of control (untransfected), GFP and miR-451 overexpressing cells showed less number of viable cells in the latter (**A**). Growth curve analysis revealed that miR-451 overexpression suppressed U2OS cell growth as compared to the control (untransfected) and GFP-expressing cells (**B**). Long term survival by colony forming assay showed reduction (60% and 40% in U2OS and Saos-2, respectively) of cell colonies in miR-451 overexpressing derivatives (**C**). Cell cycle analysis revealed increase in number of cells at G0/G1 phase and decrease in number of cells at G2/M and S phases (**D**). Tumor growth assays in subcutaneous xenograft of control (untransfected) and miR-451 overexpressing cells showed reduced tumor forming capacity of the miR-451 derivatives as compared to the control cells (**E**). Body weight of the mice during the course of experiment showed no difference (**E**).

Downregulation of miR-451 has been reported in several kinds of cancers in many recent studies^16,20,27,34^. By miR-array we detected miR-451 as an upregulated miR in cells that emerged from 5-AZA-dC-induced senescence in treated cultured cells as compared to the control. In light of this data, we performed real-time PCR assay in a variety of cancer and normal cells. Consistent with other reports, we found that all cancer cells possessed low level (~2–6 fold) of expression of miR-451 as compared to the normal cells (Fig. 1). Of note, *in vitro* immortalized lung fibroblasts (MRC5/hTERT) and lung tumor-derived cells (A549) showed lower level of expression as compared to the normal lung fibroblasts (MRC5) (Fig. 1). Furthermore, amongst several cancer cells, U2OS showed the lowest level of miR-451 expression (Fig. 1). We next overexpressed miR-451 in U2OS cells, and examined their proliferation rate with respect to the control (untransfected) and GFP-vector transfected cells. As shown in Fig. 2A, compared to the untransfected control and GFP-transfected cells, miR-451 overexpressing derivatives showed decrease in viability (Fig. 2A) and cell growth (Fig. 2B). Similar effect was observed in long-term survival and colony forming capacity (Fig. 2C and Supplementary Figure 1A). Cell cycle analysis of control and miR-derivatives revealed G0/G1 arrest of in the latter (Fig. 2D). In order to confirm that the effect was specific to miR-451, we recruited two other miRs (miR-101 and −558) using identical vector and cell line. Whereas miR-101 and miR-558 promoted cell proliferation, as determined by short term (cell viability) and long term (cell growth and colony formation) assays, miR-451 caused inhibition (Supplementary Figure 1B–D). However, contrary to our expectation, growth arrest in miR-451 transfected cells were observed also in the presence of 5-AZA-dC (Supplementary Figure 1E). The data suggest that miR-451-induced growth arrest may prevent incorporation of 5-AZA-dC in cell genome and contribute to fast recovery of cells during subsequent culture in 5-AZA-dC free medium. In order to firmly confirm the growth inhibitory activity of miR-451, we performed *in vivo* tumor progression assays using subcutaneous xenograft model. As shown in Fig. 2E and F, significant growth retardation of miR-451-overexpressing A549 derivatives as compared to the control (untransfected cells) demonstrated that it is a tumor suppressor miR.

### Tumor suppressor activity of miR-451 is mediated by upregulation of p21^WAF1^, but not p53

**Figure 3:**
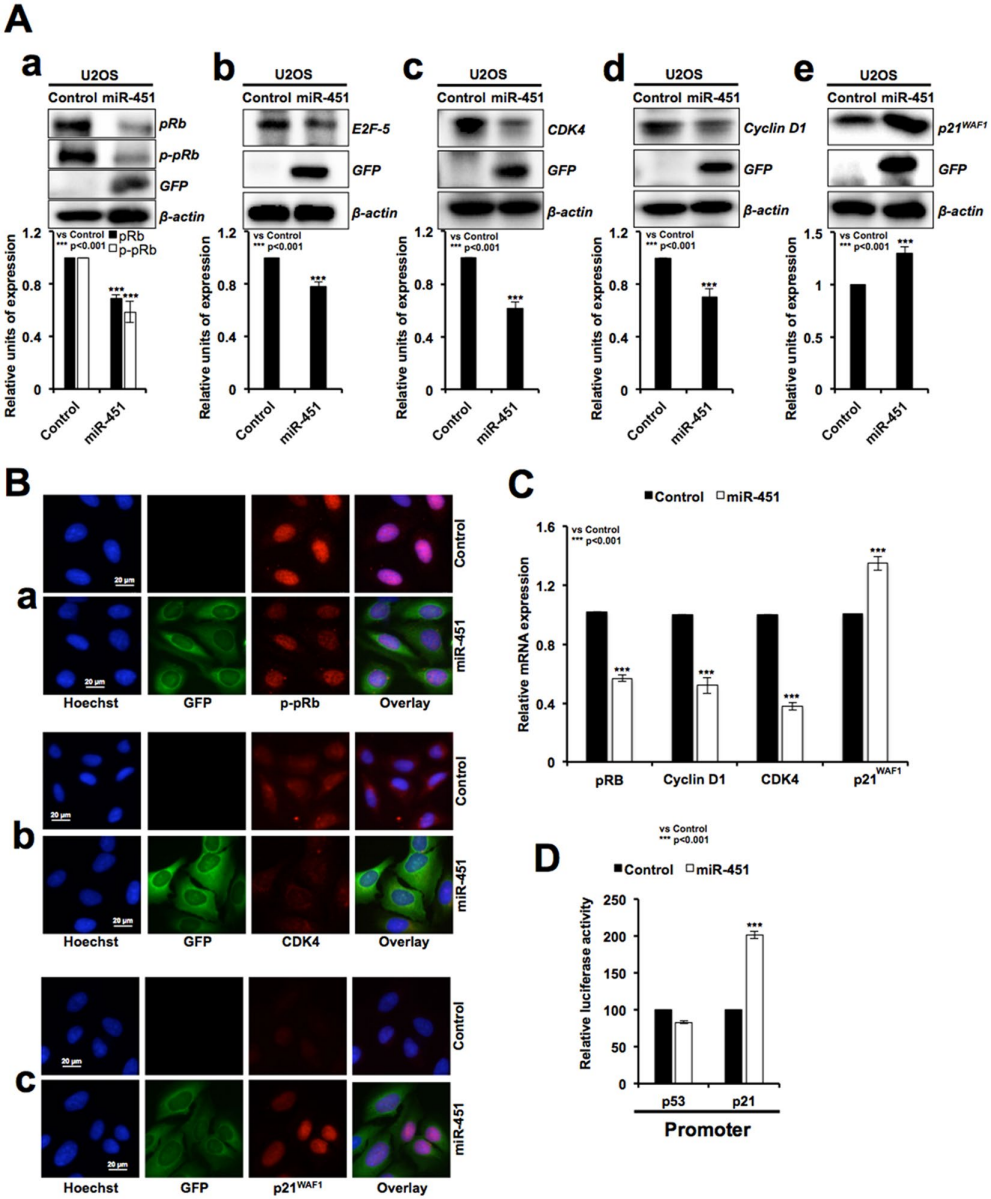
miR-451 overexpression mediated growth arrest involves upregulation of pRB and p21^WAF1^ tumor suppressor pathways. miR-451 overexpressing derivatives that showed growth arrest possessed lower level of expression of pRB, phospho-pRB, E2F-5, CDK4 and cyclin D1, and higher level of expression of p21^WAF1^ as compared to the control (untransfected) cells and determined by Western blotting (**A**) and immunostaining (**B**). mRNA expression analyses revealed, decrease in the level of pRb, cyclin D1, CDK4 and increase in p21^WAF1^ in miR-451 overexpressing cells (**C**). p53 dependent promoter driven-reporter assays in control (untransfected) and miR-335 transfected cells showed upregulation of p21^WAF1^ and no change in p53 (**D**).

**Figure 4:**
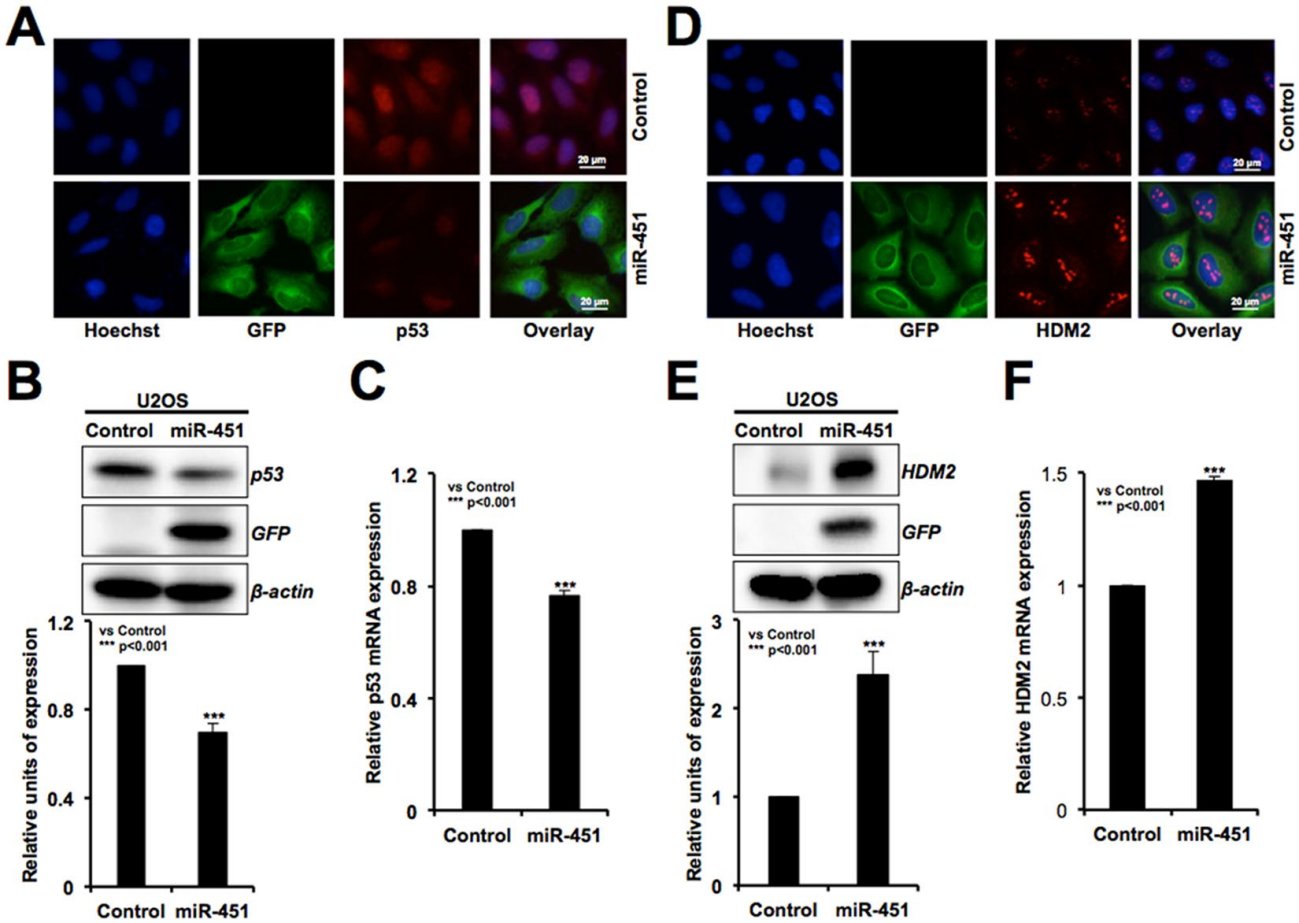
miR-451 overexpressing cells showed downregulation of p53 and upregulation of HDM2. miR-451 overexpressing derivatives possessed lower level of expression of p53 protein as well as mRNA levels (**A–C**). HDM2 was upregulated in miR-451 overexpressing as compared to the control (untransfected) cells at both mRNA and protein levels (**D–F**).

In order to investigate the mRNA targets and clarify the mechanism of miR-451-mediated growth suppression, we performed expression analyses of major tumor suppressor proteins. As shown in Fig. 3A(a–e), miR-451 overexpressing cells (as identified by GFP fluorescence), showed decrease in pRB, its phosphorylated form and E2F-5 (Fig. 3A,a,b) signifying cell cycle arrest. Consistent with these, upstream regulators of pRB-phosphorylation, CDK4 and Cyclin D1, showed decrease (Fig. 3A,c,d), in line with an increase in expression level of p21^WAF1^ (an established mediator of growth arrest and inhibitor of cyclin/CDK complexes^35^) (Fig. 3A–e). These data were supported by immunofluorescence assay with specific antibodies (Fig. 3B,a–c). We performed real time RT-PCR for these candidate target genes, and found that in agreement with the protein expression data, mRNA for pRB, Cyclin D1 and CDK-4 was decreased in miR-451 derivatives (Fig. 3C). p21^WAF1^ on the other hand, showed increase that was also supported by p21^WAF1^ promoter-luciferase assays (Fig. 3D). Contrary to increase in p21^WAF1^, p53 specific promoter-luciferase assays did not real revealed significant change in the miR-451 derivatives suggesting that p21^WAF1^ increase might be p53-independent (Fig. 3D). We examined the expression of p53 (its major transcriptional activator) and indeed found its decrease, both at the protein (Fig. 4A,B) as well as mRNA levels (Fig. 4C), in miR-451 derivatives. In line with this, HDM2 (antagonist for p53) protein and mRNA showed increase (Fig. 4D–F) that may account for decrease in p53.

### miR-451 targets CARF and results in an upregulation of p21^WAF1^

**Figure 5:**
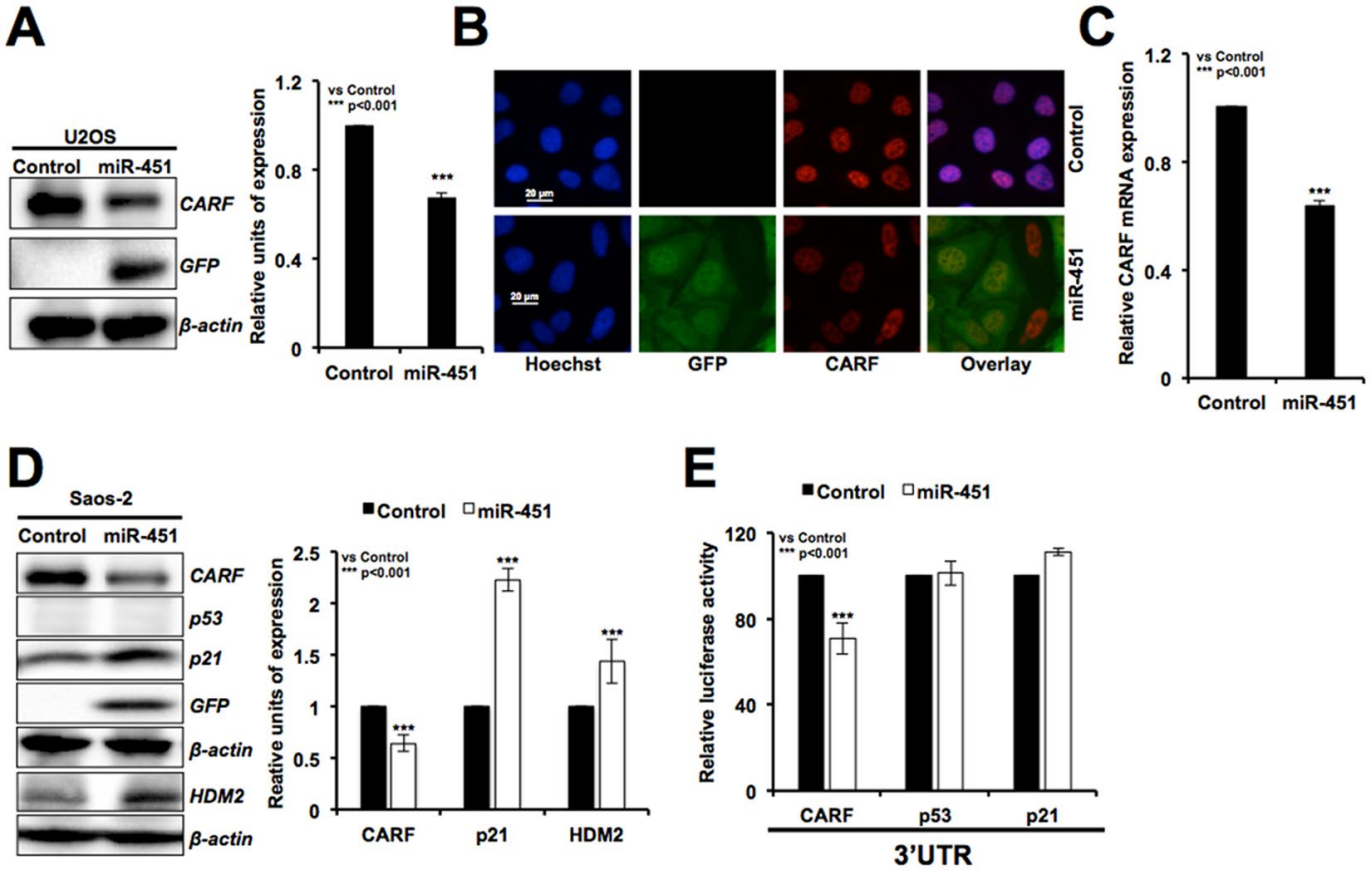
miR-451 directly targets CARF, but not p53. Western blotting (**A**) immunostaining (**B**) and qRT-PCR (**C**) analysis for CARF showed decreased level of expression in miR-451 overexpressing as compared to the control (untransfected) cells. Increase in p21^WAF1^ and HDM2 occurred in p53−/− (Saos-2) cells (**D**). 3′UTR reporter assay for CARF, p21^WAF1^, p53 in control and miR-451 overexpressing cells showed that miR-451 directly targets CARF (**E**).

We next explored the mechanism of miR-451 induced growth arrest that was marked by increase in p21^WAF1^ and hypo-phosphorylation of pRB. We have earlier reported that CARF (Collaborator of ARF) acts as a transcriptional repressor of HDM2 and p21^WAF1^. Based on the above results on upregulation of both p21^WAF1^ and HDM2 in miR-451 derivatives, we predicted that CARF could be one of the targets of miR-451. Analyses of protein as well as mRNA expression of CARF in control, vector and miR-451 derivatives revealed its remarkable decrease in the latter (Fig. 5A–C and Supplementary Figure 1F). Furthermore, increase in p21^WAF1^, HDM2 and decrease in CARF was observed in p53 deficient Saos-2-miR 451 derivative cells (Fig. 5D) suggesting that increase in p21^WAF1^ was independent to that of p53, and CARF is a candidate new target for miR-451. For conclusive validation, we performed reporter assays using CARF, p53 and p21 3′UTR-luciferase constructs. As shown in Fig. 5E, miR-451 caused reduction in CARF, and not p53 or p21^WAF1^ mRNA. These data confirmed that miR-451 targets CARF, and not p53 or p21^WAF1^.

### CARF targeting by miR-451 and miR-335 induce apoptosis

**Figure 6:**
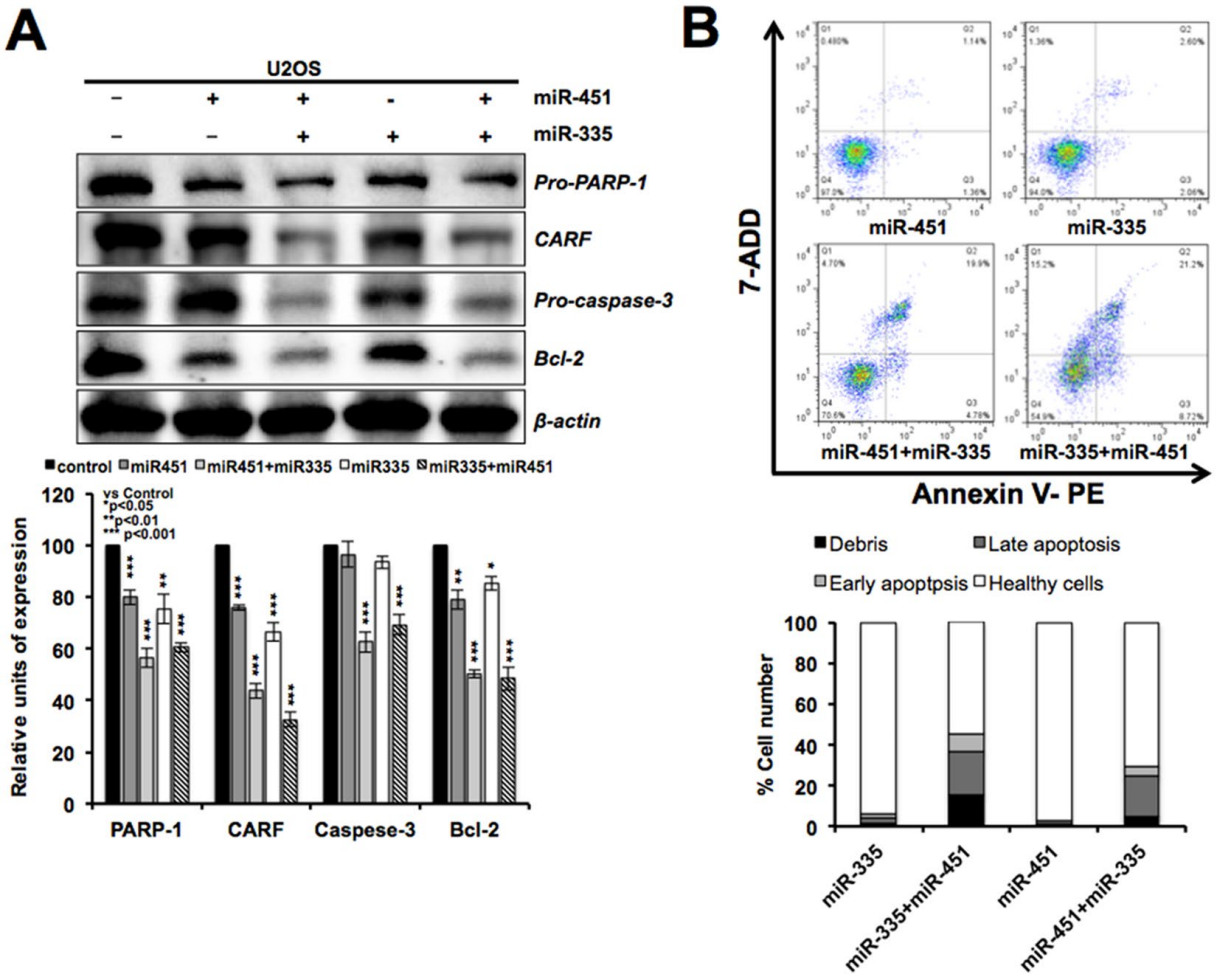
miR-335 and miR-451 co-transfection caused stronger knockdown of CARF resulting in apoptosis of cells. miR-4335 and miR-451 double derivatives showed stronger knockdown of CARF resulting in decrease in pro-caspase-3, pro-PARP-1 and Bcl-2 (**A**) and apoptosis (**B**).

CARF has been shown to pose two-way control on cell proliferation^36–40^. Its knockdown was lethal for cells *in vitro* and *in vivo* suggesting that it is an essential protein for cell survival^40^. A variety of cellular senescence models endorsed that the overexpression of CARF caused growth arrest of cancer cells and plays a definitive role in replicative as well as stress-induced senescence^36,39–41^. Super-high level of CARF expression has been associated with malignant transformation of cancer cells. Molecular mechanism(s) on the role of CARF in such dual regulation of cell proliferation has not been resolved as yet. In loss-of-function screening to identify miRNAs involved in 5-Aza-dC-induced senescence, we earlier identified miR-335 that was confirmed to target CARF^33^. It was shown that miR-335 caused growth arrest in cancer cells and hence inhibited incorporation of 5-Aza-dC in genome^33^. In view of this, we co-transfected the cells with miR-451 and miR-335 and examined the cell phenotype. As shown in Fig. 6A, cells co-transfected with miR-335 and miR-451 showed stronger knockdown of CARF resulting in apoptosis as confirmed by apoptotic markers including cleavage of procaspase-3 and decrease in anti-apoptotic protein, Bcl-2 (Fig. 6A). FACS analysis of control (untransfected) and double (miR-335 and 451)-transfected cells also confirmed higher rate of apoptosis in the latter (Fig. 6B).

**Figure 7:**
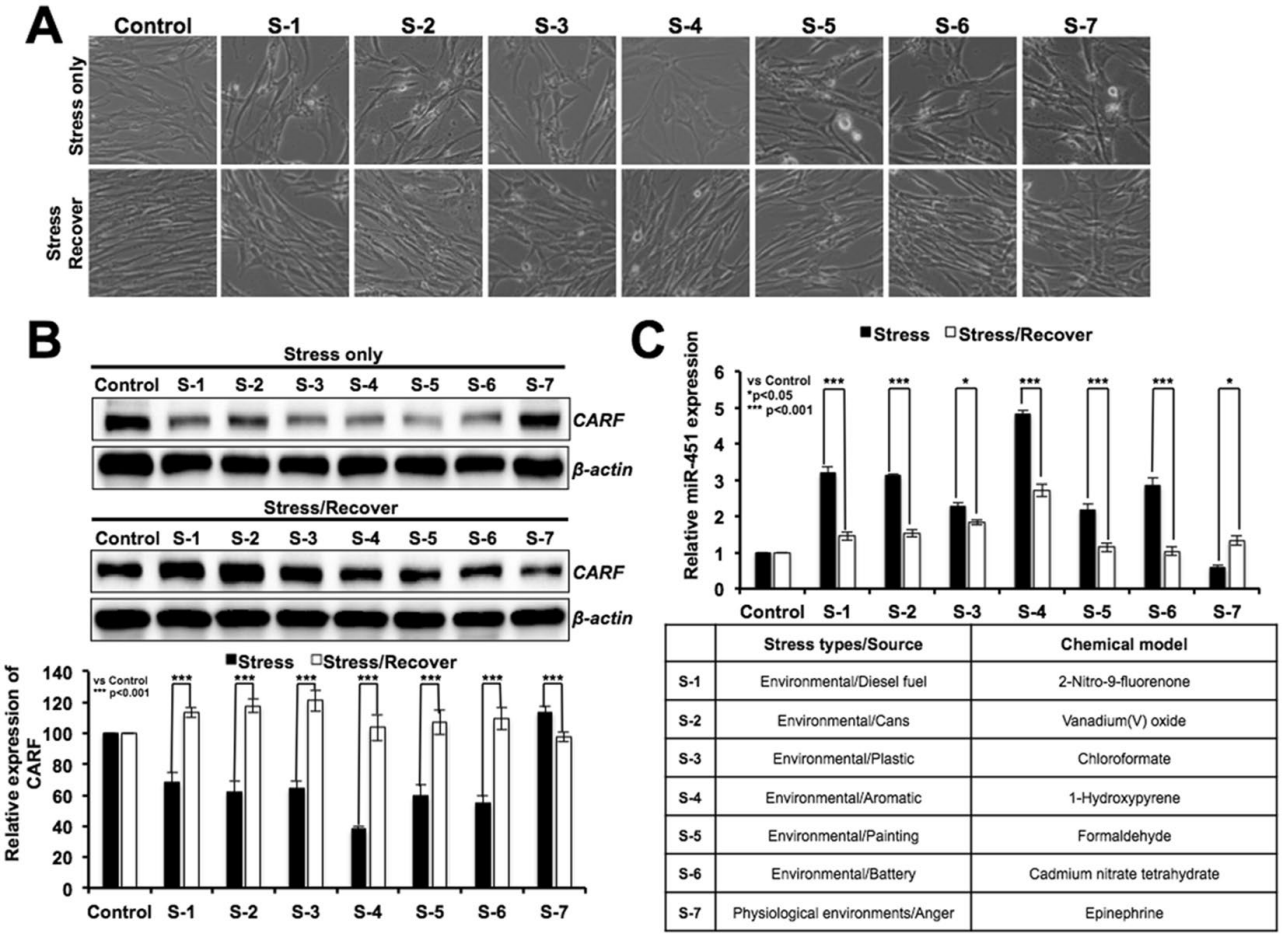
miR-451 regulates stress response of cells through CARF signaling. Phenotype of human fibroblasts under normal and a variety of stressed conditions (as shown in the table) (**A**). Western blotting showing decrease in CARF associated with apoptosis of stressed cells and its recovery in cells treated with a herbal extract (**B**). miR-451 expression was upregulated in stressed as compared to the untreated control cells, and showed downregulation in recovered cells (**C**).

In order to further prove the regulation of CARF by miR-451, we recruited seven chemical stress models that represented a variety of environmental stresses (Fig. 7A–C). Normal human fibroblasts (TIG-3) were treated with stress inducing agents that caused apoptosis, endorsed by decrease in CARF (Fig. 7A and B). Of note, consistent with the decrease in CARF, miR-451 showed increase (Fig. 7C). Furthermore, we found that the cells treated with herbal extract were protected from apoptosis. They showed recovery in CARF expression. Of note, increase in CARF was accompanied by decrease in miR-451 in stress-recovered cells (Fig. 7A–C). These data supported that miR-451 regulates CARF as well as stress response of cells.

## Discussion

Collaborator of ARF (CARF) was first reported as a binding partner and collaborator of ARF (p14^Alternate Reading Frame^ protein) by yeast two-hybrid screening^42^. It has been shown to regulate proliferative fate of cells; cellular senescence, apoptosis and malignant transformation by dose dependent two-way regulatory pathways^36–40^. It has been established that CARF is essential for cell survival; its knockdown causes apoptosis. Furthermore, whereas overexpression of CARF was shown to be associated with senescence related growth arrest of cells, its super high level of expression was correlated with malignant transformation of cancer cells^38^. Consistent with these findings, CARF has been shown to be upregulated in a variety of cancer cells^40,43^ and involved in cancer metastasis^20^. Recently we reported that the tumor suppresser miR-335 directly targets CARF and regulates cell cycle^33^.

In the present study, we recruited retroviral vector constituting two long terminal repeat (LTR) promoters on the 5′ and 3′ ends of the gene. Upon random integration of the vector in the genome, it resulted into expression of GFP; detected by green fluorescence. At the same time, it caused integration-dependent arbitrary manipulation of the host cell genome leading to altered expression of either proteins or miRs, and thereby loss-of-function phenotype, escape from induction of senescence in cancer (U2OS) cells by 5-Aza-dC (demethylating drug). The cells that evaded senescence were selected and subjected to microRNA array^33^. We found that several miRNAs (miR-101, miR-143, miR-335, miR-145, miR-451, miR-545 and miR-558) were upregulated in vector-transduced cells that showed resistance to 5-Aza-dC-induced senescence^33^. We earlier demonstrated that miR-335 was regulated by methylation and silenced in a variety of tumors. Similar to miR-335, miR-451 caused growth suppression *in vitro* and *in vivo*. Furthermore, targeting of CARF by both miR-451 and miR-335 was confirmed by double transfections of cells that caused stronger knockdown of CARF (Fig. 6). We found that miR-451-induced growth arrest was mediated by increase in p21^WAF1^ and consequent decrease in Cyclin D1, CDK4, pRB^Phospho^ and E2F5. These changes were independent of p53 status of the cells and mediated by targeting CARF, as endorsed by protein, mRNA and 3′UTR reporter assays. Increase in CARF has earlier been reported in response to stress and replicative senescence^36,37,39,40^. We found that miR-451 plays role in such regulation of CARF. It showed increase in response to a variety of stresses that caused decrease in CARF (Fig. 7). Of note, when cells were protected against stress by treatment with a herbal extract, both miR-451 and CARF showed some recovery to the normal unstressed levels suggesting a tight correlation of miR-451 with CARF. In order to further examine miR-451-driven regulation of CARF, we performed sequence based analyses using TargetSpy, a knowledge based online portal comprising broad-range compositional, physical, and combinatorial base-pairing algorithms that predicted miR-451 target sequence in CARF/CDKN2AIP 3′UTR transcript (NM_017632) located at 448–465 base position (Supplementary Figure 1G). Taken together, we demonstrate that CARF is a new target of miR-451 that mediates its tumor suppressor function in normal and stressed biological states.

## Materials and Methods

### Cell culture

All human cancer and normal cells were purchased from Japanese Collection of Research Bioresources (JCRB), Japan. Human osteosarcoma (U2OS, MG-63 and Saos-2), colorectal adenocarcinoma (DLD-1 and SW620) breast adenocarcinoma (MCF7), lung carcinoma (A549), immortalized lung fibroblasts (MRC5/hTERT) and normal human fibroblast (TIG-3 and MRC5) were cultured in Dulbecco’s modified Eagle’s medium (DMEM; Gibco BRL, Grand Island, NY, USA) supplemented with 10% (v/v) fetal bovine serum (Gibco BRL), and 1% (v/v) penicillin/streptomycin in the presence of 5% CO_2_ at 37 °C.

### RNA extraction, reverse transcription and real-time reverse transcription- polymerase chain reaction

Total RNA was isolated and quantitated from cells using the Relia Prep RNA Cell Miniprep System (Promega, Madison, WI, USA) and NanoDrop ND-1000 (Nanodrop Technologies, Wilmington, DE, USA), respectively. Pure RNA with A260/A280 ratio >1.9 was used for reverse transcription using QuantiTect Reverse Transcription Kit (Qiagen, Hilden, Germany) following the manufacturer’s instructions. The cDNA thus obtained was used for real-time qRT-PCR (50 °C for 2 min; 95 °C for 10 min, 40 cycles of 95 °C for 15 s, 60 °C for 1 min; and 72 °C for 30 s) using SYBR®Select Master Mix (Applied Biosystems, CA, USA) in triplicate on the EcoTM real time system (lllumina, San Diego, CA, USA). The results of real-time qRT-PCR were normalized to the geometric mean of the 18 s internal control gene for variability in expression levels, then calculated as 2^−[(Ct, Target gene − Ct, 18s) Time x −(Ct, Target gene − Ct, 18s) Time 0]^ following the manufacturer’s instructions, where Ct represents the threshold cycle for each transcript. The sequence of primers used is listed in Table 1.

**Table 1:** qRT-PCR primer set sequences.

Gene (human)	Sequence (5′-3′)
p21^WAF1^ forward	GAGGCCGGGATGAGTTGGGAGGAG
p21^WAF1^ reverse	CAGCCGGCGTTTGGAGTGGTAGAA
Cyclin D1 forward	TCCTCTCCAAAATGCCAGAG
Cyclin D1 reverse	GGCGGATTGGAAATGAACTT
CDK4 forward	TCGAAAGCCTCTCTTCTGTG
CDK4 reverse	TACATCTCGAGGCCAGTCAT
pRb forward	GGAAGCAACCCTCCTAAACC
pRb reverse	TTTCTGCTTTTGCATTCGTG
p53 forward	TAACAGTTCCTGCATGGGCGGC
p53 reverse	AGGACAGGCACAAACACGCACC
HDM2 forward	TAGTATTTCCCTTTCCTTTGATGA
HDM2 reverse	CACTCTCCCCTGCCTGATAC
CARF forward	TCAAAGTGACAGATGCTCCA
CARF reverse	CGTTGAACTGTTTTCCTGCT
18s forward	CAGGGTTCGATTCCGTAGAG
18s reverse	CCTCCAGTGGATCCTCGTTA

In order to detect the expression level of miR-451 in cells, the corresponding miR-451 (Assay ID: 464419 mat) and RNU6B (Assay ID: 001093) primers and TaqMan® MicroRNA assay were performed following manufacturer’s instructions (Applied Biosystems, CA, USA). Real-time qRT-PCR was performed at 95 °C for 10 min, 40 cycles of 95 °C for 15 s and 65 °C for 1 min. The miR-451 expression data were normalized to the endogenous control based on the threshold cycle (C_t_) calculated as 2^−[(Ct, miR-451 − Ct, RNU6B) Time x −(Ct, miR-451 − Ct, RNU6B) Time 0]^. Stem loop sequence of miR-451 and RNU6B are showed in Table 2.

**Table 2:** Stem loop sequence.

miR-451	UGGGUAUAGCAAGAGAACCAUUACCAUUACUAAACUCAG UAAUGGUAACGGUUUCCUUGCCAUUCCCA
RNU6B	GCAAGGATGACACGCAAATTCGTGAAGCGTTCCATATTTTT

### Cloning of miR-451, expression plasmid and transfection

pCXGb-miR-451 encoding GFP and miR-451 driven by chicken β-actin promoter was generated by amplification of miR-451 from human genomic DNA by PCR using following primers: Sense 5′AAAGTCGACAAGCTCTCTGCTCAGCCTGTC3′ and antisense 5′AAAATATCTCGAGCCCCCACCCCTGCCTTGT3′. The PCR product was digested with *Sal*I and *EcoR*V and introduced into pCXGb plasmid as described earlier^33^.

Cells were transfected with miR-451 plasmid using X-remeGENE HPDNA transfection reagent (Roche Applied Science, Indianapolis, USA). Typically, 1 μg and 5 μg of miR-451 plasmid were used for 6-well dish and 10-cm dish of cells at around 70% confluency, respectively. The transfection efficiency was determined by GFP fluorescence. Vector containing GFP encoding, without miR, sequence was used as an empty control.

### Cell viability, proliferation and colony forming assays

For short-term cell viability, 5 × 10^3^ control (untransfected), vector-transfected and miR-451 transfected cells were plated in 96-well plates, incubated at 37 °C for 48 h followed by addition of MTT (100 μl, 5 mg/ml in PBS) (Sigma-Aldrich, Missouri, USA) to each well and further incubation at 37 °C for 4 h. The supernatant was replaced with 100 μl of dimethyl sulfoxide (DMSO) and the chromophore was quantitated at 570 nm, using microplate reader (Infinite M200 PRO, TRCAN).

For cell proliferation assay, equal number of control (untransfected), vector-transfected and miR-451 transfected cells were plated in 12-well plates. Cultured cells were harvested at indicated time points. The viable cells were counted by trypan blue exclusion assay using TC20^TM^ Automated Cell Counter (Bio-Rad, Hercules, CA, USA). Growth curves were generated for each cell line from three independent experiments.

Effect of miR-451 on long-term proliferation of cells was determined by colony forming assay. Control (untransfected) or transfected cells (500/well) were plated in 6-well plates. The cells were cultured for next two weeks with regular (every third day) change of culture medium until colonies appeared. Colonies were fixed with methanol/acetone (1/1, v/v) for 10 min at 4 °C, stained with 0.1% Crystal violet for overnight, de-stained with water. Plates were dried and photographed using scanner (EPSON). Colonies were counted and statistical analysis was performed as described below.

### Flow cytometry analysis

Equal number of control (untransfected) and transfected cells were seeded in 10-cm dishes. After 24 h of seeding, cells were cultured in serum free medium for 24 h followed by harvesting using 0.25% trypsinization. Cell pellets were washed with cold PBS and then added, drop by drop into the pre-cooled 70% ethanol to fix the cells. The fixed cells were stored at −20 °C for 24 h to until further use. The cells were centrifuged at 500 × *g* for 5 min, washed with cold PBS twice, re-suspended in 1 ml cold PBS and were treated with RNase A (100 μg/ml) at 37 °C for 1 h to avoid false DNA-PI staining. RNase A-treated cells were centrifuged to discard the supernatant. The pellet was re-suspended in 200 μl of Cell Cycle Guava reagent (Millipore, Billerica, MA, USA), mixed gently and incubated for 30 min in dark. The stained cells were subjected to cell cycle analysis using Guava PCA flow cytometer (Millipore), and FlowJo Software (version 7.6, Flow Jo, LLC, USA).

### Apoptosis assay

Control (untransfected) U2OS (2 × 10^5^) and their miR-451 or miR-335^33^ derivatives were seeded in 6-well plates. Twelve hours later, the cells were transfected with either miR-335 or miR-451 using X-remeGENE HPDNA transfection reagent. Cells were cultured for 48–72 h cells and were then harvested by trypsin (0.25%) and cells centrifuging at 500 × *g* for 10 min at 4 °C. Cells were re-suspended in medium to make the cells number between 2 × 10^5^ ml. 100 μl of cells were incubated with 100 μl of Guava Nexin Reagents (Millipore, Billerica, MA, USA) for 20 min in dark, and analyzed by Guava PCA flow cytometer (Millipore). The data were further analyzed by using FlowJo Software (version 7.6, Flow Jo, LLC, USA).

### Luciferase reporter assay

pMIR-CARF-3′UTR plasmid was used as described earlier^33^. The pGL4-p53-3′UTR and pGL4-p21-3′UTR were generously provided by Dr. Chae-Ok Yun (Hanyang University, Seoul, South Korea). Equal number of control (untransfected) and transfected cells were plated in 6-well plates. Cells were transfected with 1 μg of luciferase constructs (pGL4-p53-3′UTR, pGL4-p21-3′UTR or pMIR-CARF-3′UTR) and 100 ng of control vector oligonucleotide (pRL-TK or pMIR-REPORTTM β-gal control plasmid) using X-remeGENE HPDNA transfection reagent (Roche Applied Science, Indianapolis, USA). Cells were harvested at 70% confluency followed by quantitation of luciferase activity using Dual-Luciferase Reporter Assay System and Tecan Infinite M200 Pro Microplate Reader (TECAN) following the manufacturer’s instructions.

### Western blotting

Cells were lysed in RIPA buffer (Thermo Scientific, Waltham, MA, USA) supplemented with a protease inhibitor cocktail (Roche). The protein concentrations were determined by using the Pierce BCA Protein Assay Kit (Thermo Scientific). 20 μg of protein for each sample were resolved in SDS-polyacrylamide gel, and electroblotted onto PVDF membranes (Millipore). Membranes were blocked in 3% BSA in TBS-T. The membranes were incubated with following primary antibodies: anti-p53 (DO-1), anti-MDM2 (HDM2-232), anti-cyclin D1 (72-13 G), anti-Cdk4 (C-22), anti-E2F-5 (MH5), anti-NFkB p65 (sc-109), anti-GFP (B-2) from Santa Cruz Biotechnology (Santa Cruz, CA, USA); anti-p21^WAF1^ (12D1), anti-Phospho-RB (Ser780), anti-Rb (4H1) from Cell signaling (Danvers, MA, USA) and anti-CARF (FL-10)^44^. Anti-β-actin antibody (AC-15) (Abcam, Cambridge, MA, USA) was used to determine the level of actin expression (as an internal loading control). All experiments were performed in triplicates. Quantitation of immunoblots was performed using the ImageJ software (National Institute of Health, Bethesda, MD).

### Immunostaining

Control (untransfected) and transfected cells (5 × 10^4^ cells/well) were seeded on 18-mm glass coverslips placed in 12-well culture dish. Cells were washed with cold PBS, fixed by using pre-cold methanol/acetone (1:1) mixture for 10 min at 4 °C and permeabilized using 0.5% Triton X-100 in PBS (PBS-T) for 10 min. The fixed cells were blocked with 0.2% BSA in PBS and were then incubated with specific primary antibodies as described above, for 1 h at room temperature or overnight at 4 °C. After washing cells with PBS-T for three times, they were incubated with Alexa-594-conjugated goat anti-mouse or anti-rabbit (Molecular Probes, Invitrogen) secondary antibodies. Nuclear staining was performed with Hoechst 33342 (Sigma) for 10 min in dark after washings (thrice) with PBS-T. Following three further washings with PBS-T, the cells were examined under Carl Zeiss microscope (Axiovert 200 M, Tokyo, Japan). Images were quantified by ImageJ software (National Institute of Health, Bethesda, MD).

### *In vivo* tumor formation assay

Four-weeks old female BALB/c nude mice were used for subcutaneous xenograft experiments. Mice (five per group) were subcutaneously injected with 5 × 10^6^ human lung cancer A549 cells (control, untransfected and miR-451 transfected cells) in 0.2 ml of PBS. Cells formed tumors in 10 days. Mice body weight; general activity (movements and eating behavior) and tumor volume were monitored every 2 days. Volume of the subcutaneous tumors was calculated as V = L × W^2^/2, where L was length and W was the width of the tumor, respectively. All animal experiments were performed following the protocols for animal experiments recognized and approved by the Animal Care and Use Committee, Institute of Laboratory Animal Science of Peking Union Medical College (ILAS-PG-2014-018).

### Statistical analysis

All experiments were carried out, at least, three times, and data were expressed as mean ± standard deviation (SD). As shown in figures, the data were with respect to control (untransfected) or vector-transfected set either at 100 or 1. Two-tailed Student’s t-test or nonparametric ManneWhitney U-test, whichever was applicable, was used to determine the degree of significance between the control and experimental sample. Statistical significance was defined as significant (*p-value ≤ 0.05), very significant (**p-value ≤ 0.01) and very very significant (***p-value ≤ 0.01).

## Electronic supplementary material

Supplementary Figures 1 and 2
