# Supplementary material for: Tumor suppressor activity of miR-451: Identification of CARF as a new target
Source: Sci Rep. 2018 Jan 10;8:375. doi: 10.1038/s41598-017-18559-5 (PMC5762681; doi:10.1038/s41598-017-18559-5)

Tumor suppressor activity of miR-451: Identification of CARF as a new target

Ling Li[1,2], Ran Gao[1,3], Yue Yu[1,2], Zeenia Kaul[4], Jia Wang[1,2], Rajkumar S. Kalra[1], Zhenya Zhang[2], Sunil C Kaul[1*] & Renu Wadhwa[1*]

[1]Drug Discovery and Assets Innovation Lab, DBT-AIST International Laboratory for Advanced Biomedicine (DAILAB), DAICENTER, Biomedical Research Institute, National Institute of Advanced Industrial Science & Technology (AIST), Tsukuba - 305 8565, Japan. [2]Graduate School of Life and Environmental Sciences, University of Tsukuba, Japan. [3]Institute of Laboratory Animal Science, Chinese Academy of Medical Science (CAMS) & Comparative Medicine Center, Peking Union Medical College (PUMC), China. [4]Department of Molecular Virology, Immunology and Medical Genetics, The Ohio State University, Columbus, Ohio 43210, USA

SUPPLEMENTARY INFORMATION

Supplementary Fig. 1 legend and Figure

Supplementary Fig. 2 legend and Full length western blots/gel displayed in main Figures 3-7 and in Suppl. Fig. 1

Supplementary Figure 1. Specificity of miR-451 in cell growth arrest assays and targeting of CARF. Viability of control, miR-101, miR-558 and miR-451 transfected cells showing decrease in number of viable cells in the latter (**A**). Growth curve analysis revealed that miR-451, not miR-101 and miR-558, suppressed cell growth (**B**). Long term survival as seen by colony forming assay showed reduction in colonies in miR-451, not miR-101 and miR-558, overexpressing derivatives (**C**). Control as well as miR-451 transfected cells showed growth arrest in response to 5-AZA-dC treatment (**D**). Target site of miR-451 in 3' UTR of CARF/CDKN2AIP is shown (**E**).

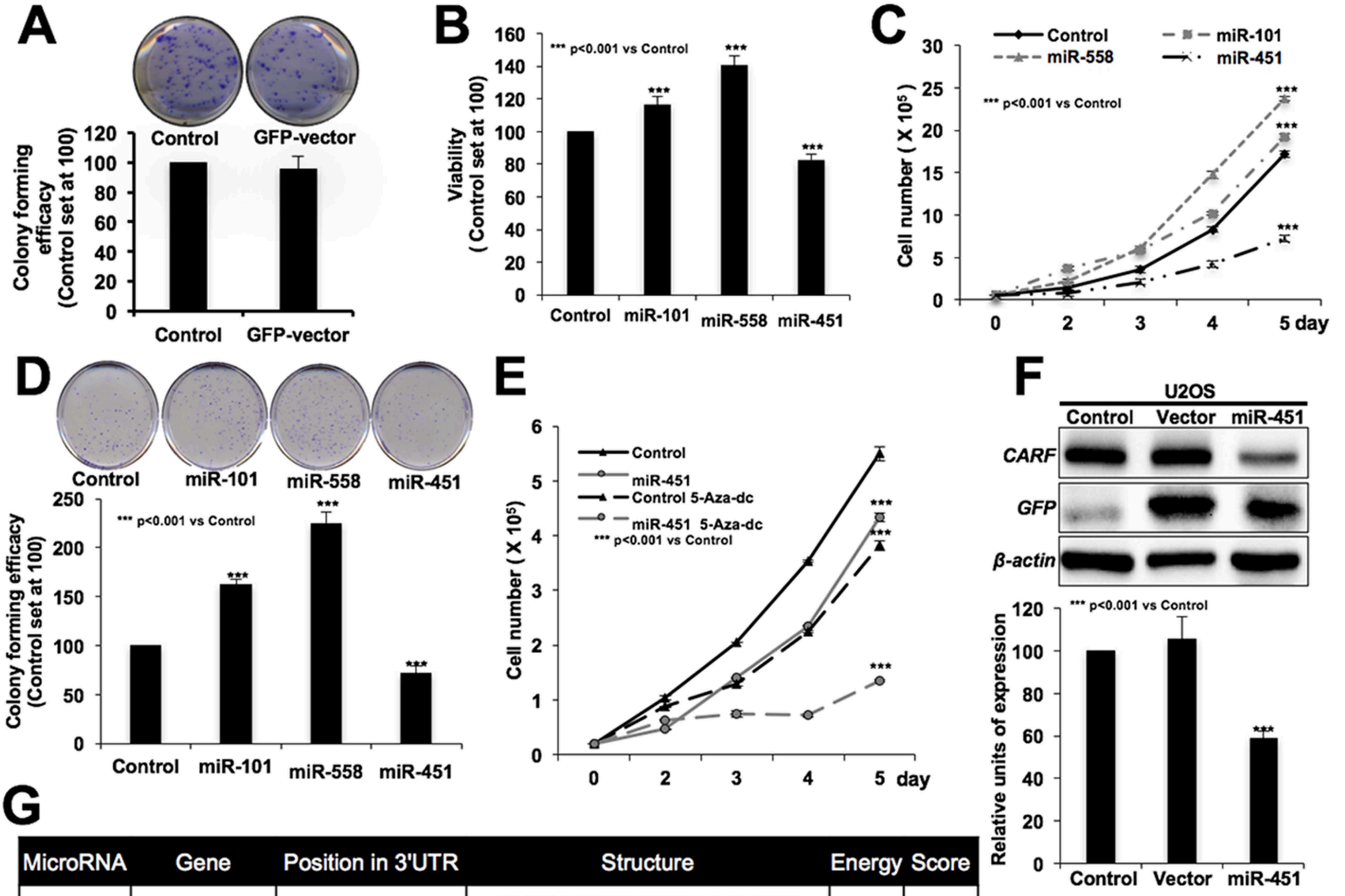

G

MicroRNA	Gene	Position in 3'UTR	Structure	Energy	Score
miR-451	NM_017632	448-465	TTGCCCGTAATGTTGAACGTGTCT<br>|||||| |||| || | UUGAGUCAUUACCA-UUGC-CAAA	-8.8	0.99

Supplementary Figure 2

Full length western blots/gel displayed in main Figures 3-7 and in Suppl. Fig. 1

Full length gel of Western blot panels in Fig. 3A-a

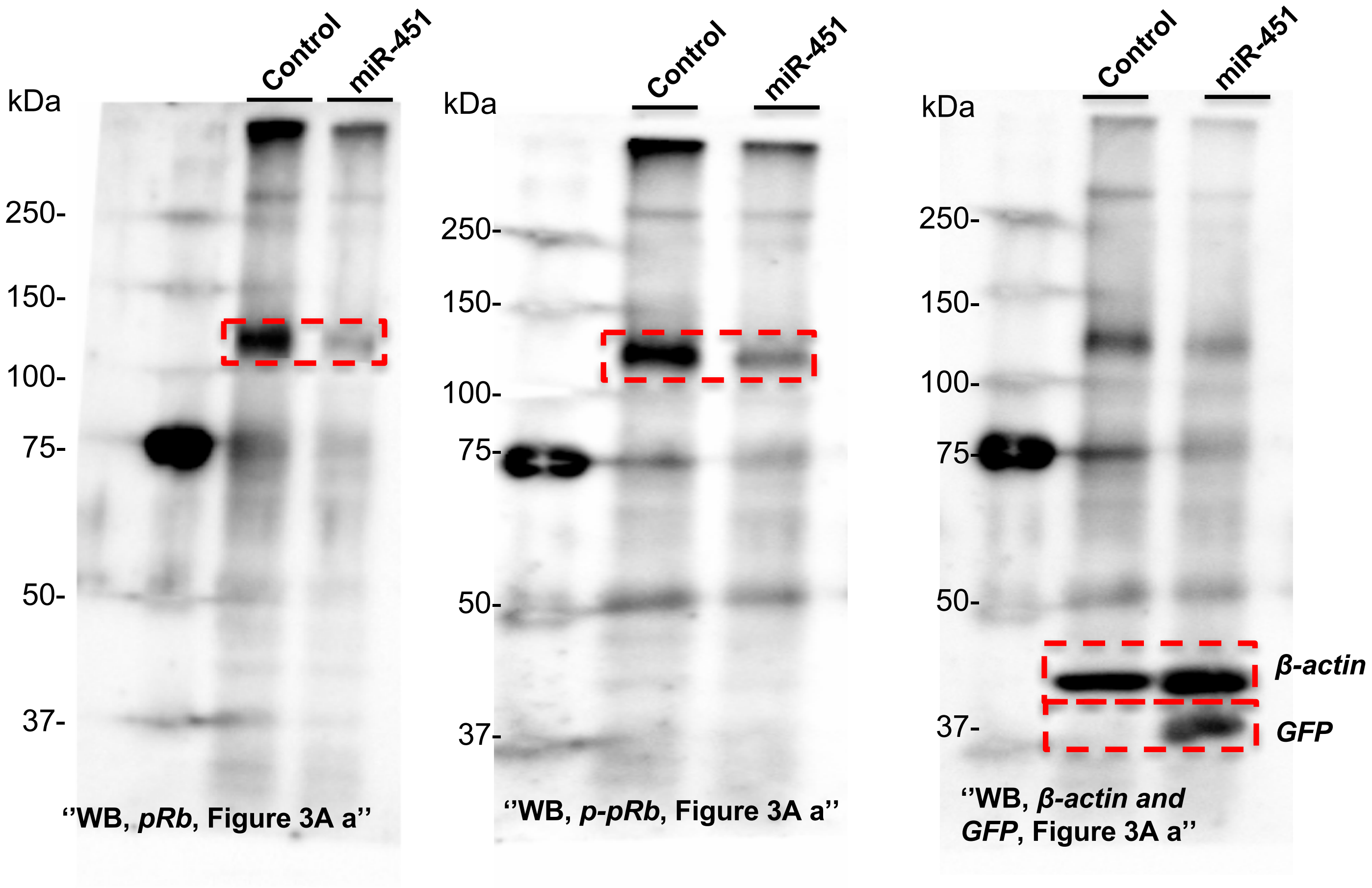

Full length gel of Western blot panels in Fig. 3A-b

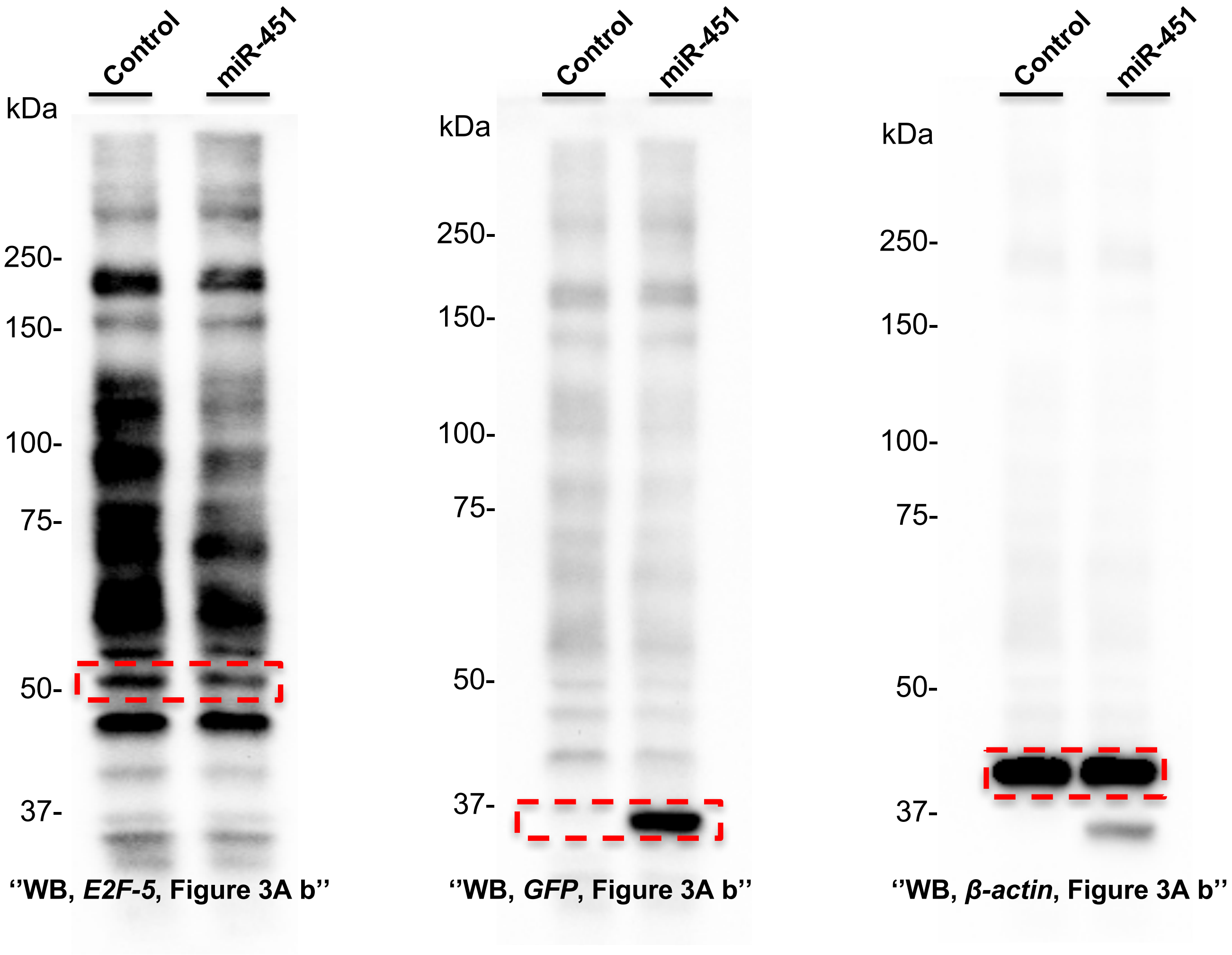

Full length gel of Western blot panels in Fig. 3A-c

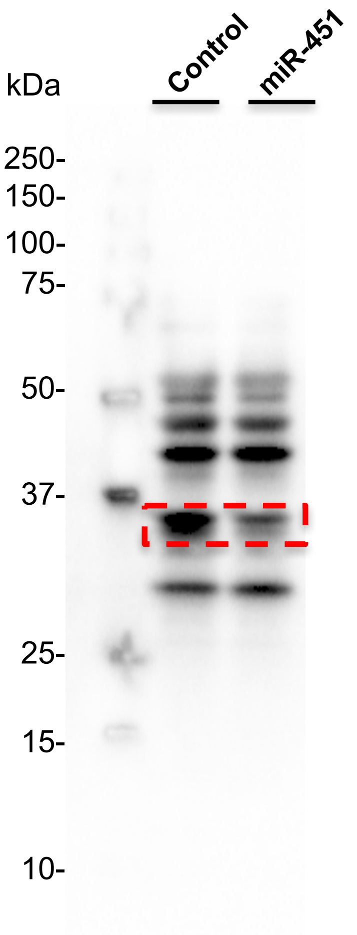

''WB, ***CDK4***, **Figure 3A c''**

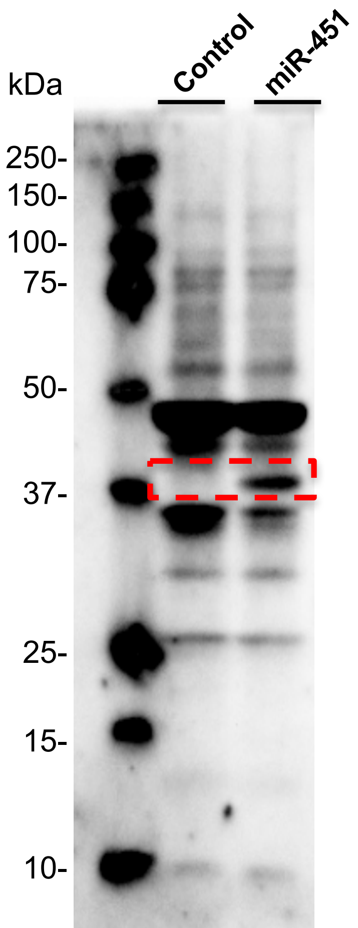

''WB, ***GFP***, **Figure 3A c''**

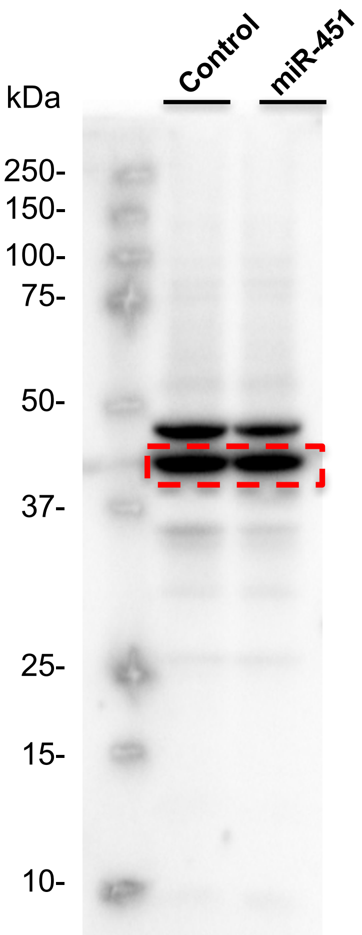

''WB, ***β-actin***, **Figure 3A c''**

Full length gel of Western blot panels in Fig. 3A-d

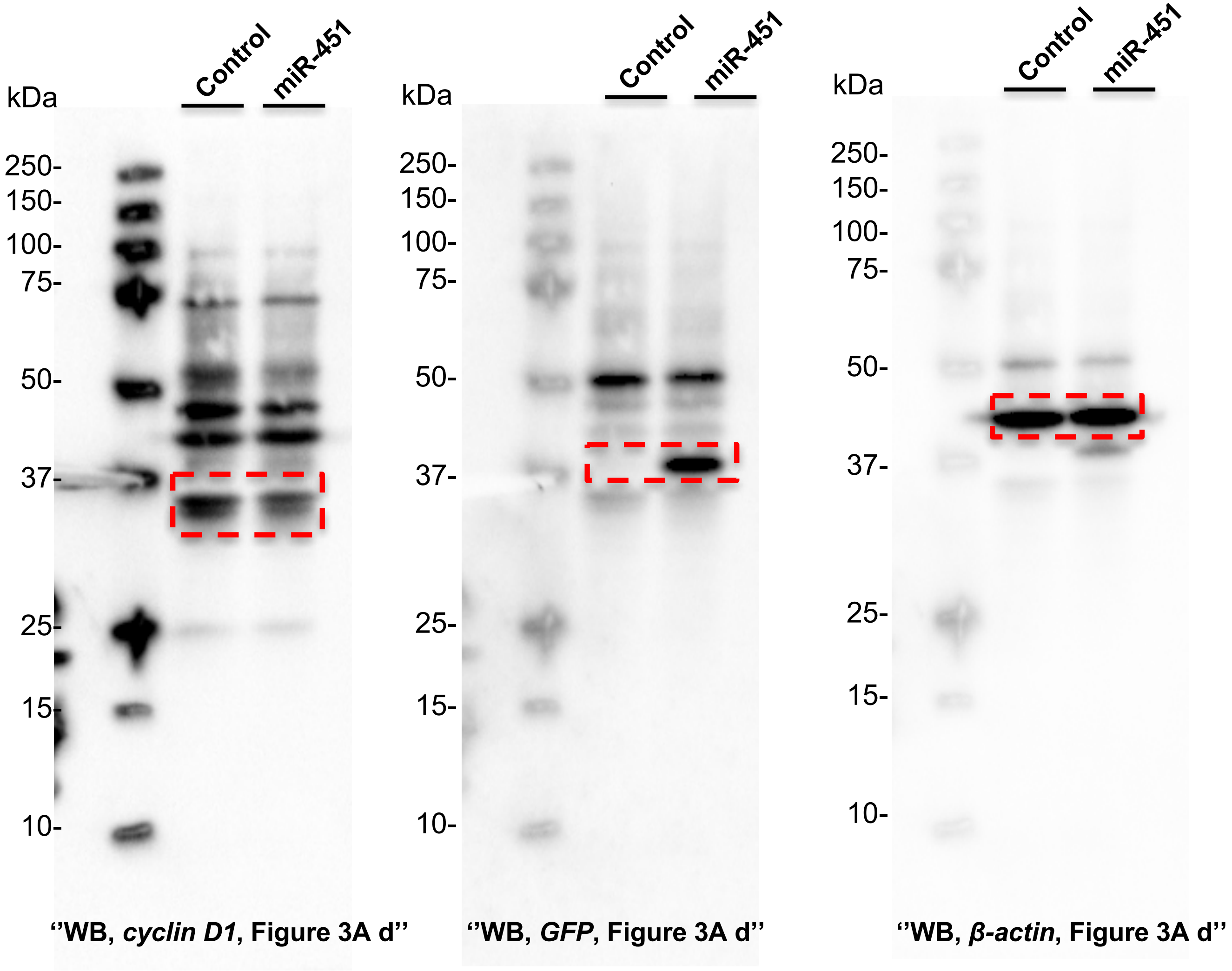

Full length gel of Western blot panels in Fig. 3A-e

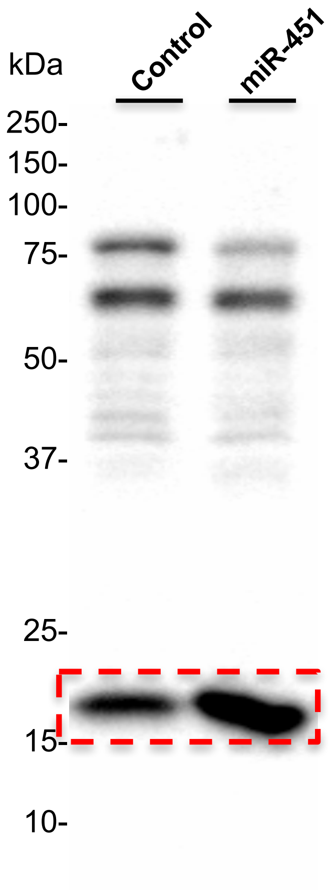

''WB, $p21^{WAF1}$
Figure 3A e''

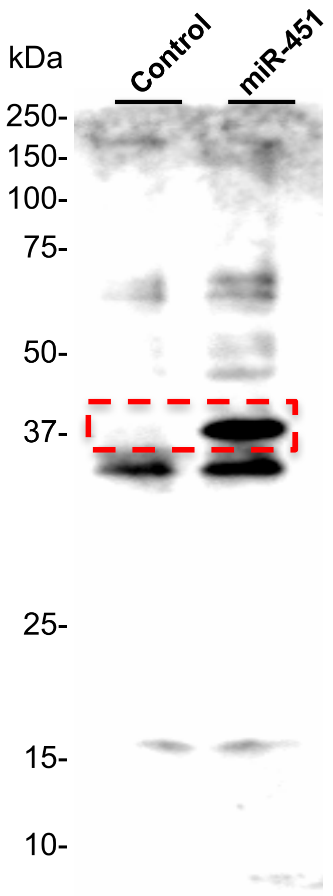

''WB, *GFP*, Figure 3A e''

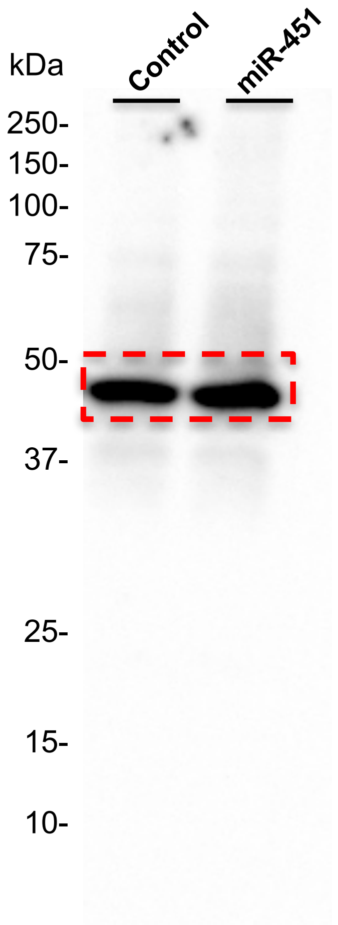

''WB, *β-actin*, Figure 3A e''

Full length gel of Western blot panels in Fig. 4B

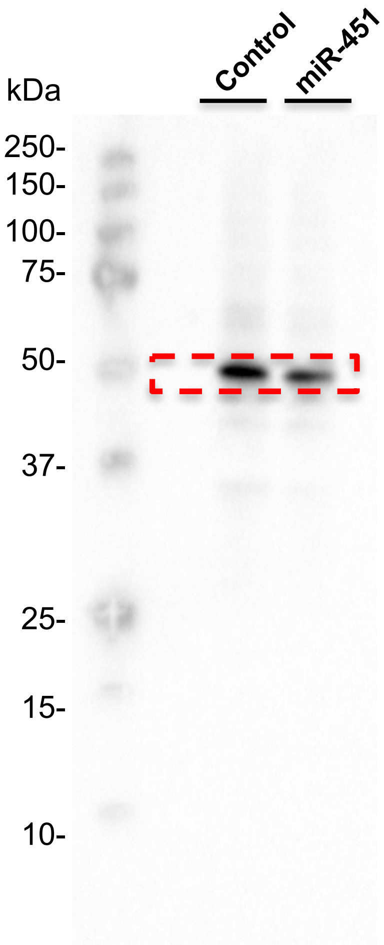

''WB, *p53*,Figure 4B''

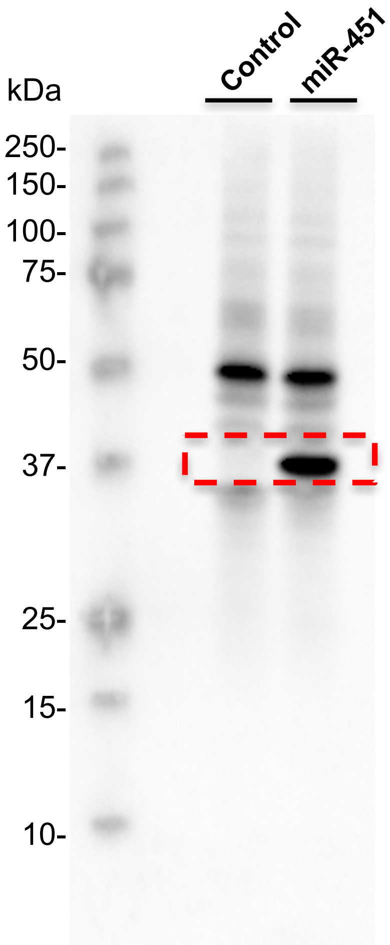

''WB, *GFP*, Figure 4B''

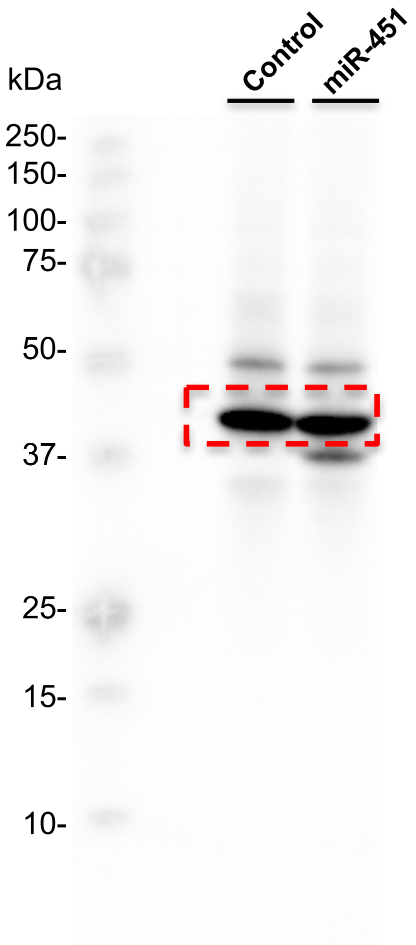

''WB, *β-actin*, Figure 4B''

Full length gel of Western blot panels in Fig. 4E

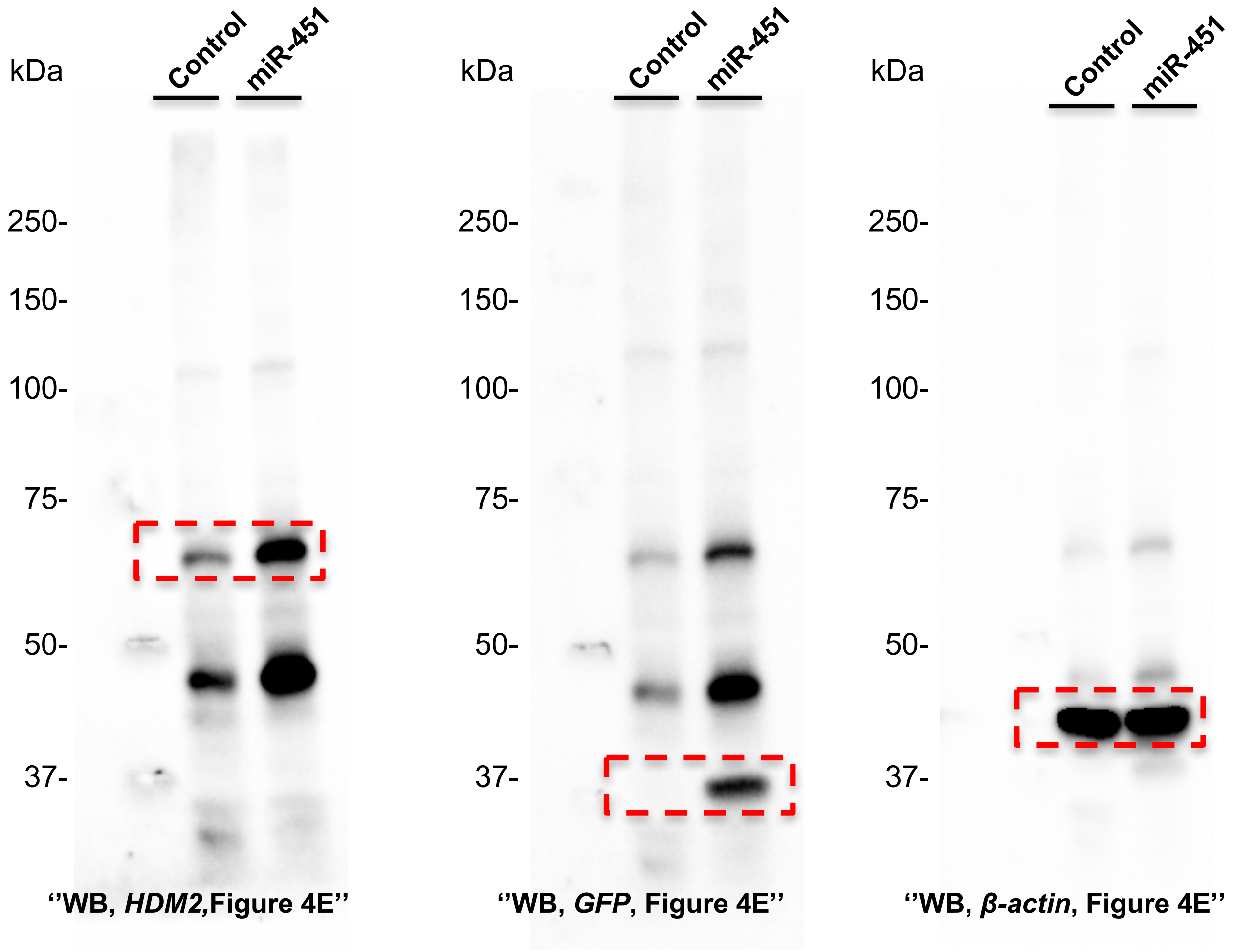

Full length gel of Western blot panels in Fig. 5A

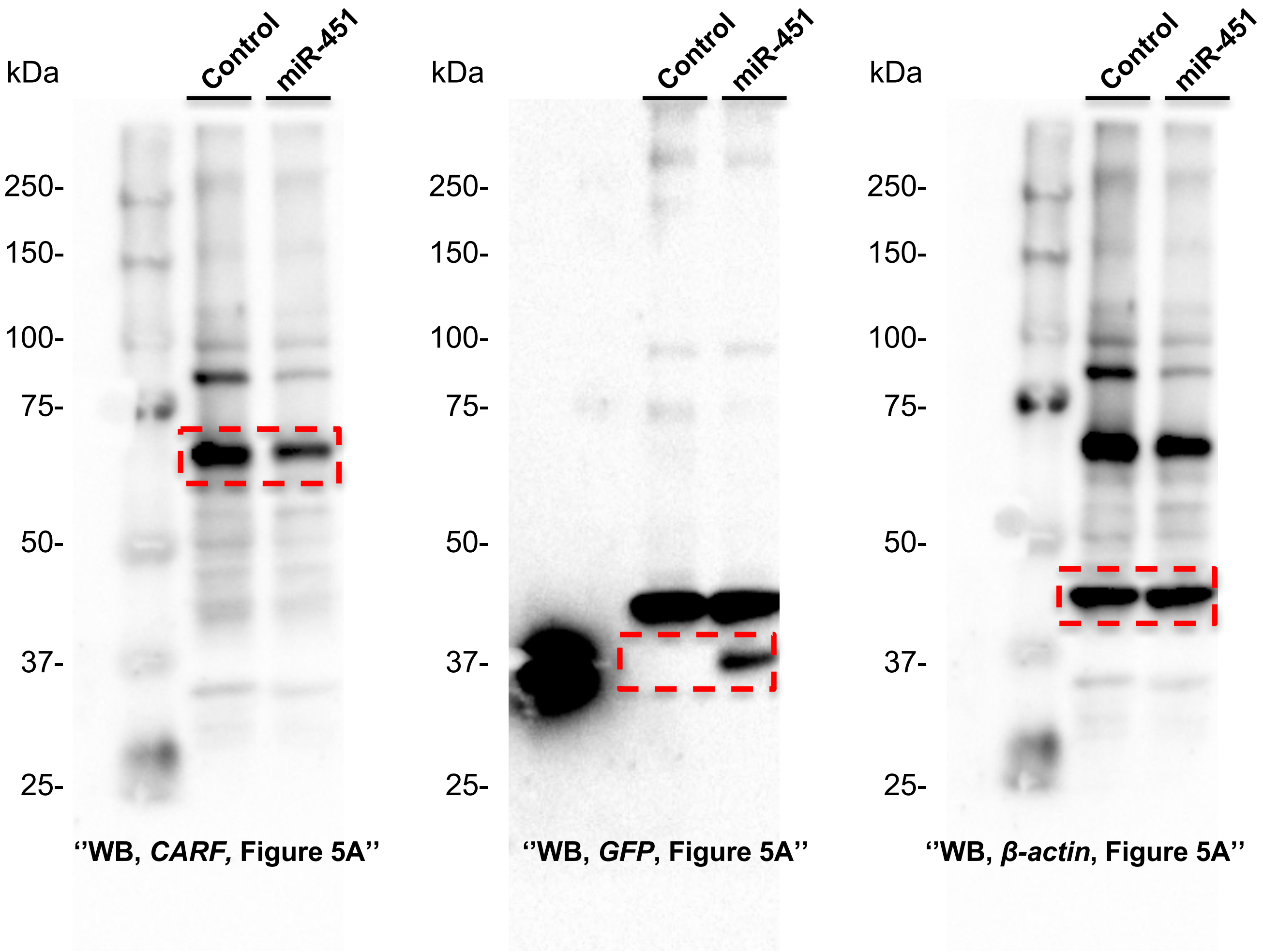

''WB, *CARF,* Figure 5A''   ''WB, *GFP*, Figure 5A''   ''WB, *β-actin*, Figure 5A''

Full length gel of Western blot panels in Fig. 5D

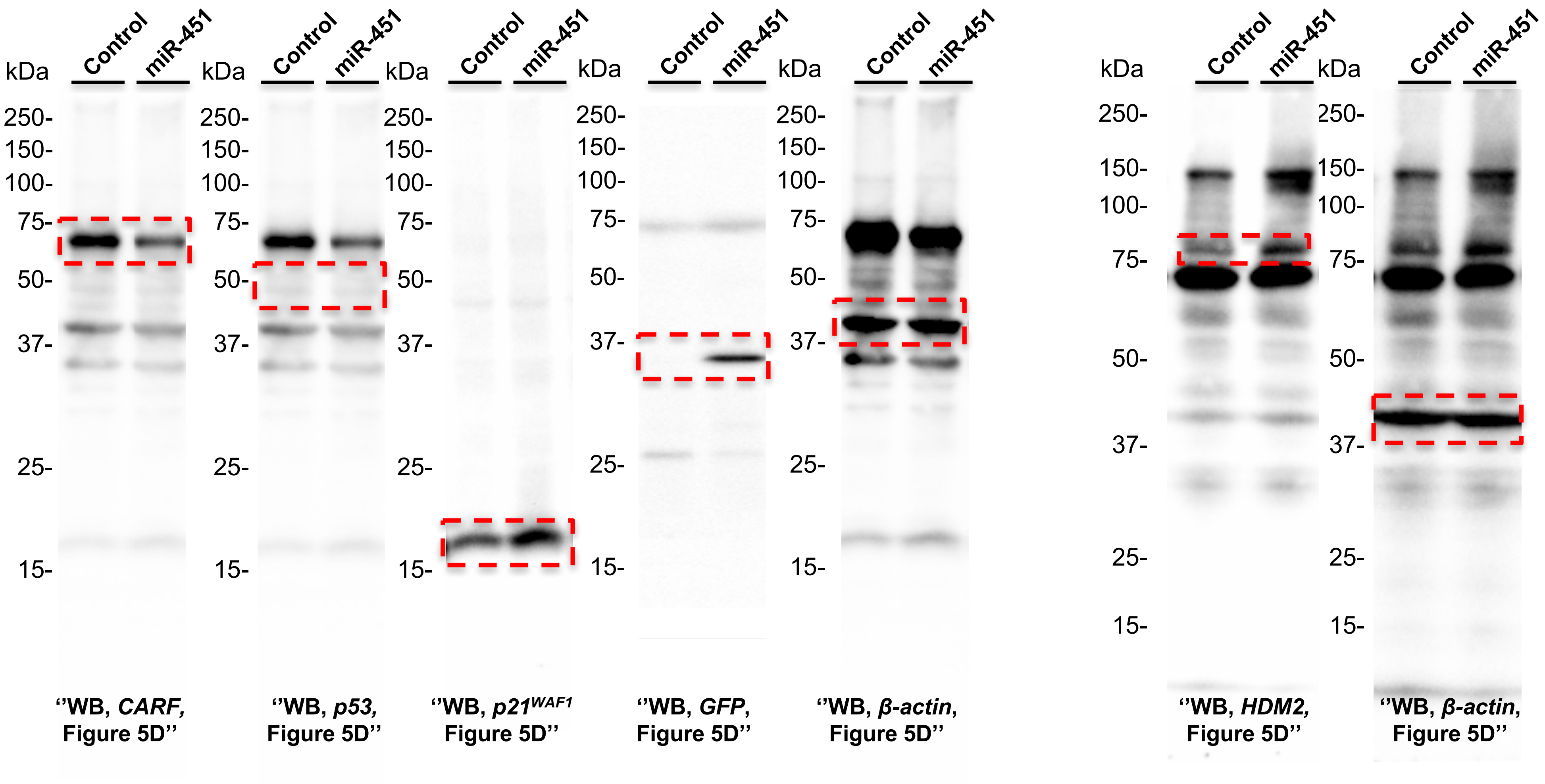

Full length gel of Western blot panels in Fig. 6A

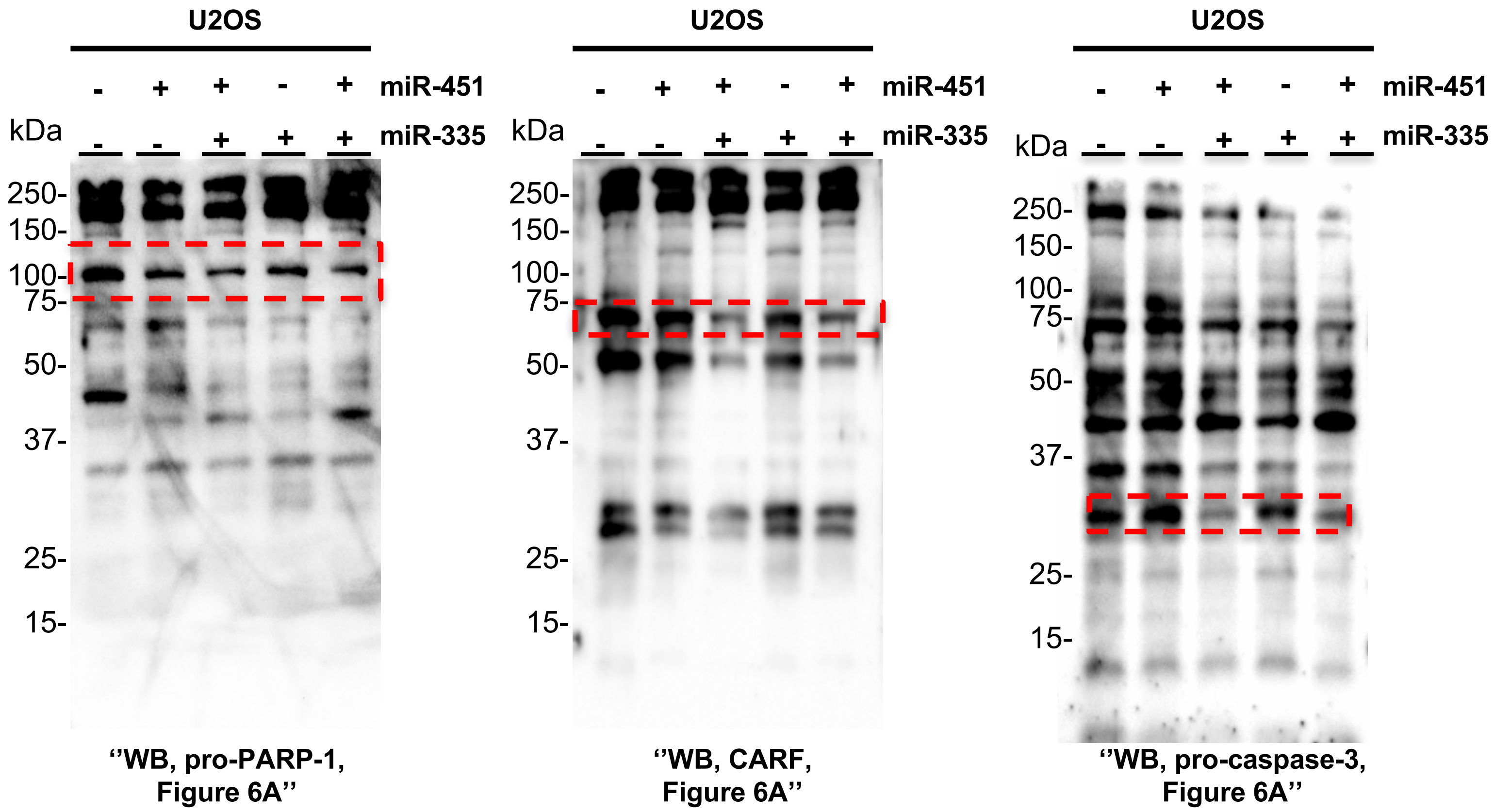

Full length gel of Western blot panels in Fig. 6A

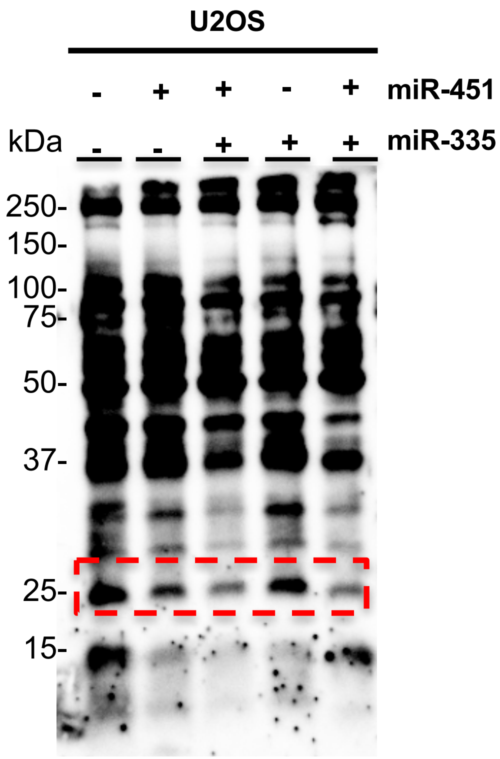

"WB, Bcl-2,
Figure 6A"

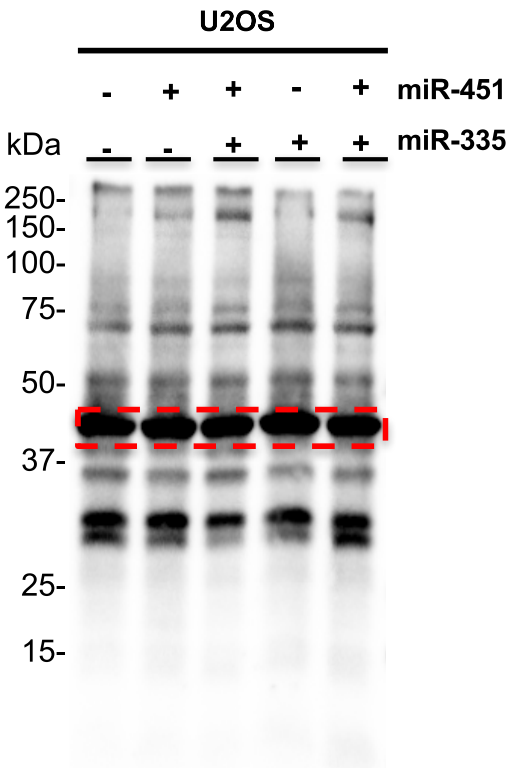

"WB, *β-actin*,
Figure 6A"

Full length gel of Western blot panels in Fig. 7B

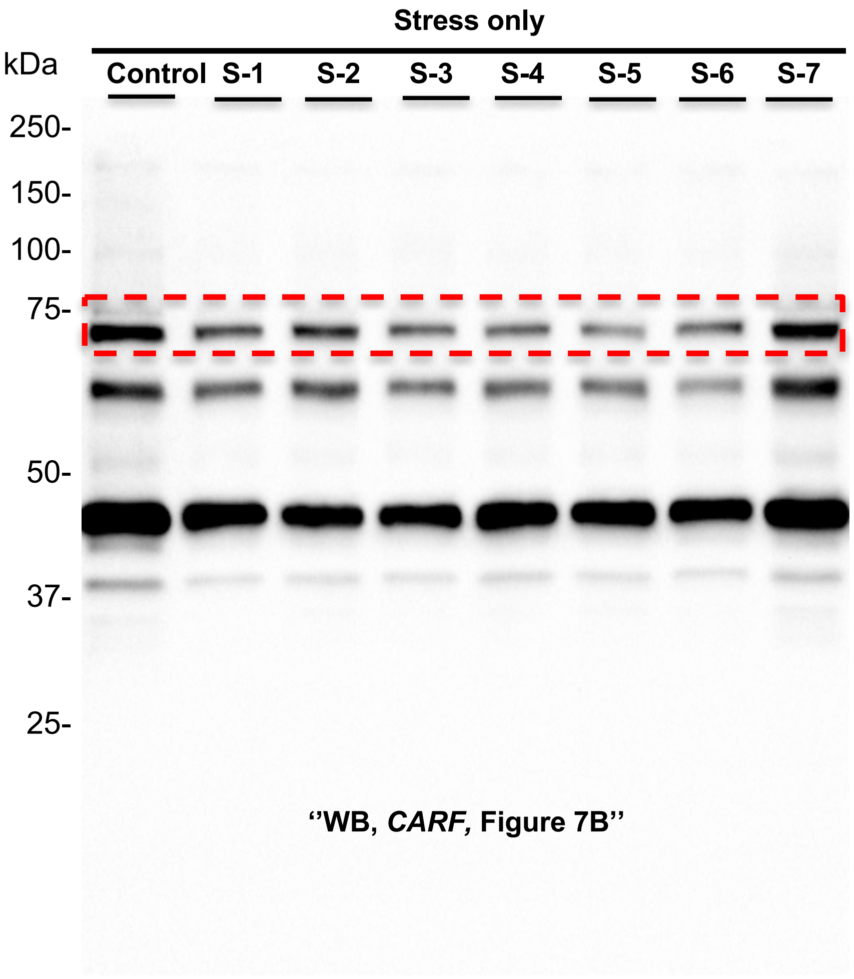

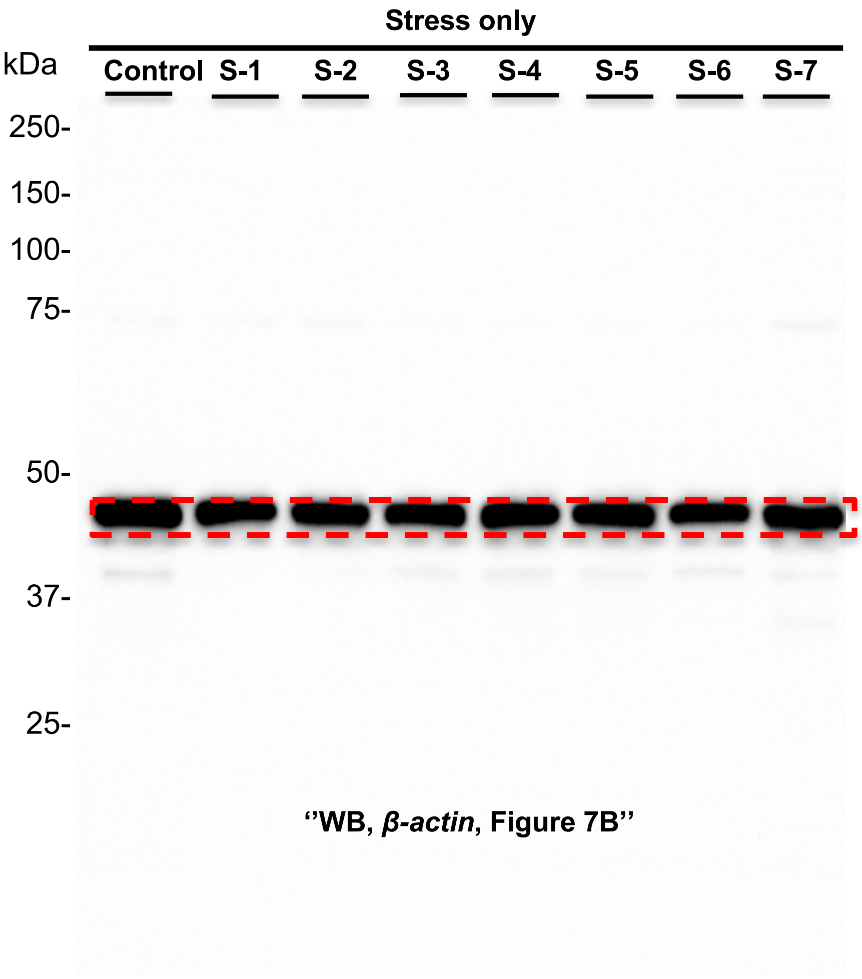

Full length gel of Western blot panels in Fig. 7B

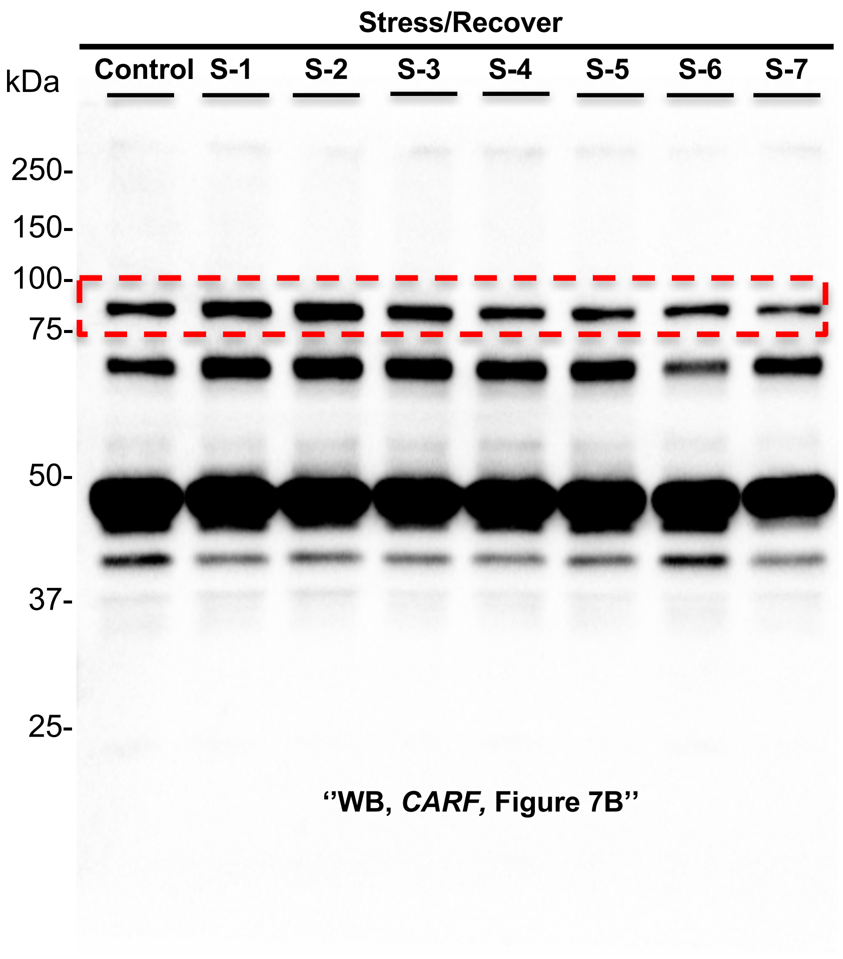

"WB, *CARF,* Figure 7B"

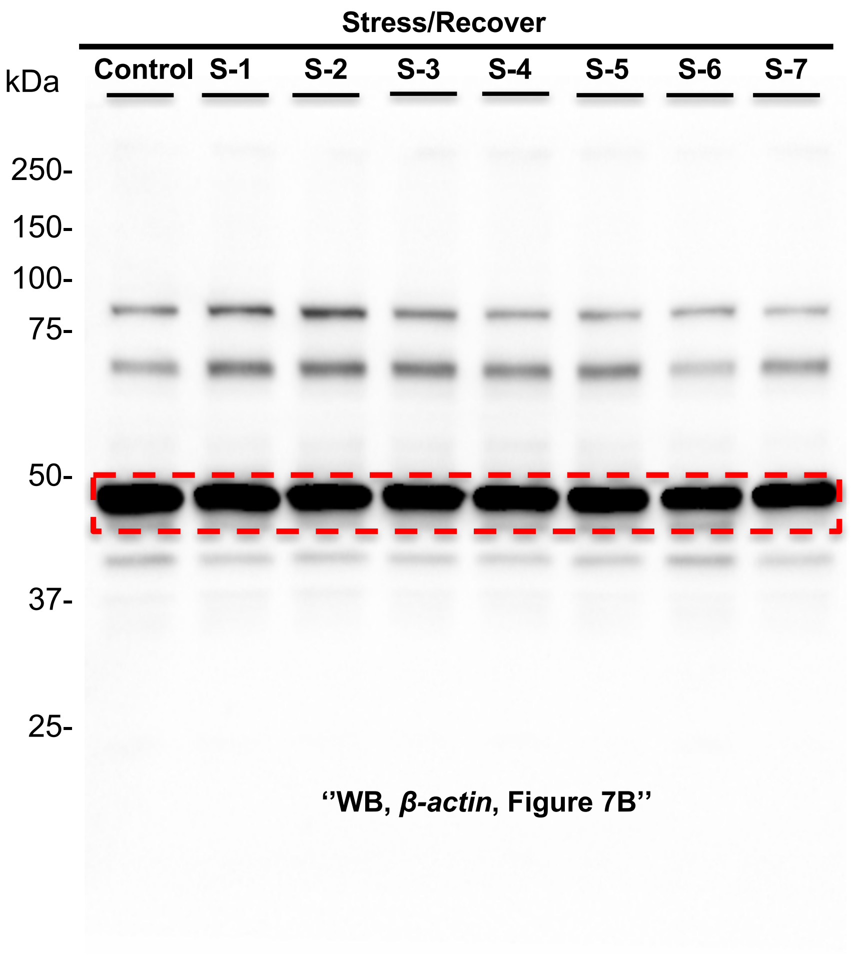

"WB, *β-actin*, Figure 7B"

Full length gel of Western blot panels in Supl. Fig. 1F

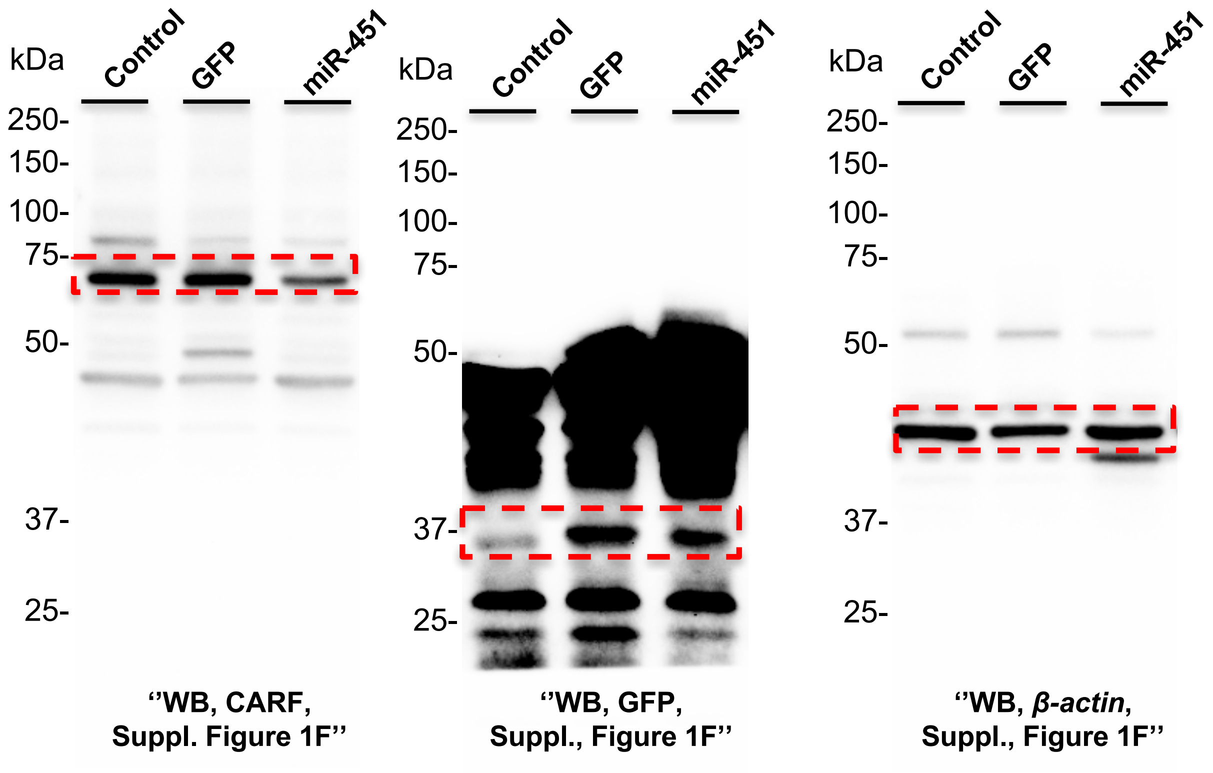

Supplement: Supplementary file 1 — Supplementary Figures 1 and 2 [file 41598_2017_18559_MOESM1_ESM.pdf]
